# Metal-Substituted Hydroxyapatite Nanoparticles as Antimicrobial and Osteogenic Biomaterials for Hard-Tissue Applications

**DOI:** 10.3390/ma19163461

**Published:** 2026-08-14

**Authors:** Ammar Z. Alshemary, Zhishang Sun, Kairui Shi, Yimeng Xu, İsmail Seçkin Çardaklı

**Affiliations:** 1 Nanomaterials Research Center, Department of Chemistry, College of Science, Mathematics and Technology, Wenzhou-Kean University, 88 Daxue Road, Ouhai, Wenzhou 325060, China; sunzhis@kean.edu (Z.S.);; 2 Dorothy and George Hennings College of Science, Mathematics and Technology, Kean University, 1000 Morris Ave, Union, NJ 07083, USA; 3 College of Science, Mathematics, and Technology, Wenzhou-Kean University, Wenzhou 325060, China; 4 Department of Metallurgical and Materials Engineering, Atatürk University, Erzurum 25240, Türkiye; cardakli@atauni.edu.tr

**Keywords:** hydroxyapatite, metal-ion substitution, antibacterial biomaterials, osteogenesis, bone tissue engineering

## Abstract

Bacterial colonization and biofilm formation on orthopedic and dental implants remain major clinical complications, while conventional systemic antibiotics are often limited by poor penetration into biofilms and infected bone. These limitations have motivated the development of biomaterials with intrinsic antibacterial activity. Hydroxyapatite (HA), a major inorganic component of bone and teeth, possesses excellent biocompatibility, osteoconductivity, and bone-bonding ability but exhibits limited inherent antibacterial activity. Incorporation of therapeutic metal ions, including Ag^+^, Cu^2+^, Zn^2+^, Ti^4+^, Co^2+^, Ga^3+^, Sr^2+^, and Ce^3+^, has therefore emerged as a promising strategy for developing multifunctional HA-based biomaterials. This review critically examines the crystal-chemical basis of metal-ion incorporation into HA and discusses how ionic radius, oxidation state, charge-compensation mechanisms, dopant concentration, and synthesis conditions influence lattice occupancy, physicochemical properties, and biological performance. The antibacterial activity of metal-substituted and metal-modified HA systems generally involves interconnected mechanisms, including bacterial membrane damage, intracellular metabolic disruption, interference with enzymes and nucleic acids, reactive oxygen species (ROS)-mediated oxidative stress, and inhibition of bacterial adhesion and biofilm formation. Ag-, Cu-, Zn-, and Ga-containing HA systems show the most consistently reported antibacterial effects, although their efficacy and cytocompatibility depend strongly on dopant concentration and ion-release kinetics. Co-substituted HA may combine antibacterial activity with angiogenic and osteogenic stimulation, whereas Sr-substituted HA is primarily osteogenic and anti-resorptive, with variable antibacterial performance. Ti-modified HA and TiO_2_/HA composites exhibit predominantly photoactive antibacterial behavior, while Ce-substituted HA shows concentration-, oxidation-state-, and synthesis-dependent biological effects. The review also evaluates protein adsorption, osteogenic and angiogenic signaling, macrophage-mediated immunomodulation, biological safety, and representative commercial and translational applications. Overall, metal-substituted HA represents a versatile platform for infection-resistant and regenerative hard-tissue biomaterials, provided that composition, phase structure, ion release, antibacterial efficacy, and cytocompatibility are systematically co-optimized before clinical translation.

## 1. Introduction

### 1.1. Calcium Phosphates and Hydroxyapatite as Biomaterials

The calcium phosphate (CP) family has many significant applications in orthopedic and biomedical engineering fields [1]. CP-based materials have received increasing attention in recent years due to their excellent similarity to natural hard tissues. Due to their good osteoconductive and non-toxic properties, they have been considered as materials of choice for various medical treatments [1,2,3]. CP-based materials are bioactive, whereas hydroxyapatite (HA) and tricalcium phosphate (TCP) are the most important members of this family and are frequently used as biomaterials. HA and TCP are well-known for their biocompatibility, osteoconductivity, osteointegration, and crystallographic and compositional similarities to the mammalian skeletal system. These CP-based compounds have attracted considerable attention due to their broad range of biomedical applications. They have been extensively used for the repair of bone, dental, and periodontal defects; alveolar bone augmentation; sinus lift procedures; and the treatment of major hard-tissue defects [4,5,6,7,8]. In addition, CP-based materials can serve as scaffolds for tissue engineering and tooth regeneration applications [8,9]. Historically, the first CP-based material was introduced as a bone substitute in the 1920s, followed later by the development and application of TCP [10].

There are many well-known compounds of the CP class; HA (Ca_10_(PO_4_)_6_(OH)_2_) is well recognized due to its exceptional bioactivity and magnificent biocompatibility with living hard tissues. Owing to its outstanding bioactivity and biocompatibility, it has played a key role in biomedical engineering, especially in bone and mineralized dental tissues, such as dental enamel and cement [8,9,11]. Furthermore, its ability to promote osteointegration and osteoconduction makes it a major inorganic component of bones and teeth and can be used in several forms, including powders, sintered porous blocks, and granules. Nanosized HA with controlled particle size and morphology is suitable for a wide range of biomedical applications, ranging from load-bearing to targeted drug delivery [12,13]. Furthermore, nanosized HA with different morphologies has been investigated for bone-defect repair and dental applications, including enamel and dentin remineralization and the treatment of dentin hypersensitivity [14]. In addition, nanosized HA has been incorporated into toothpastes and other oral-care products to promote enamel remineralization, reduce the depth of early carious lesions, and facilitate the formation of a new apatite layer [14].

### 1.2. Crystal Structure of Hydroxyapatite and Metal-Ion Substitution

In contrast to stoichiometric synthetic HA, biological apatite, the main inorganic component of hard tissues, is usually carbonated and contains trace amounts of ions such as F^−^, Cl^−^, SiO_4_^4−^, Na^+^, Mg^2+^, K^+^, Sr^2+^, Zn^2+^, Ba^2+^, and Al^3+^ [15]. These minor ionic substitutions are important because they influence the crystallinity, solubility, biological response, and overall performance of bone minerals. Although synthetic HA is widely used in biomedical applications, its biological and mechanical performance may be limited due to the absence of certain naturally occurring ionic components [15]. In addition, pure HA has low mechanical strength and limited intrinsic antibacterial activity, which limits its direct use in load-bearing applications and increases the risk of bacterial adhesion and biofilm formation after implantation [16].

**Figure 1:**
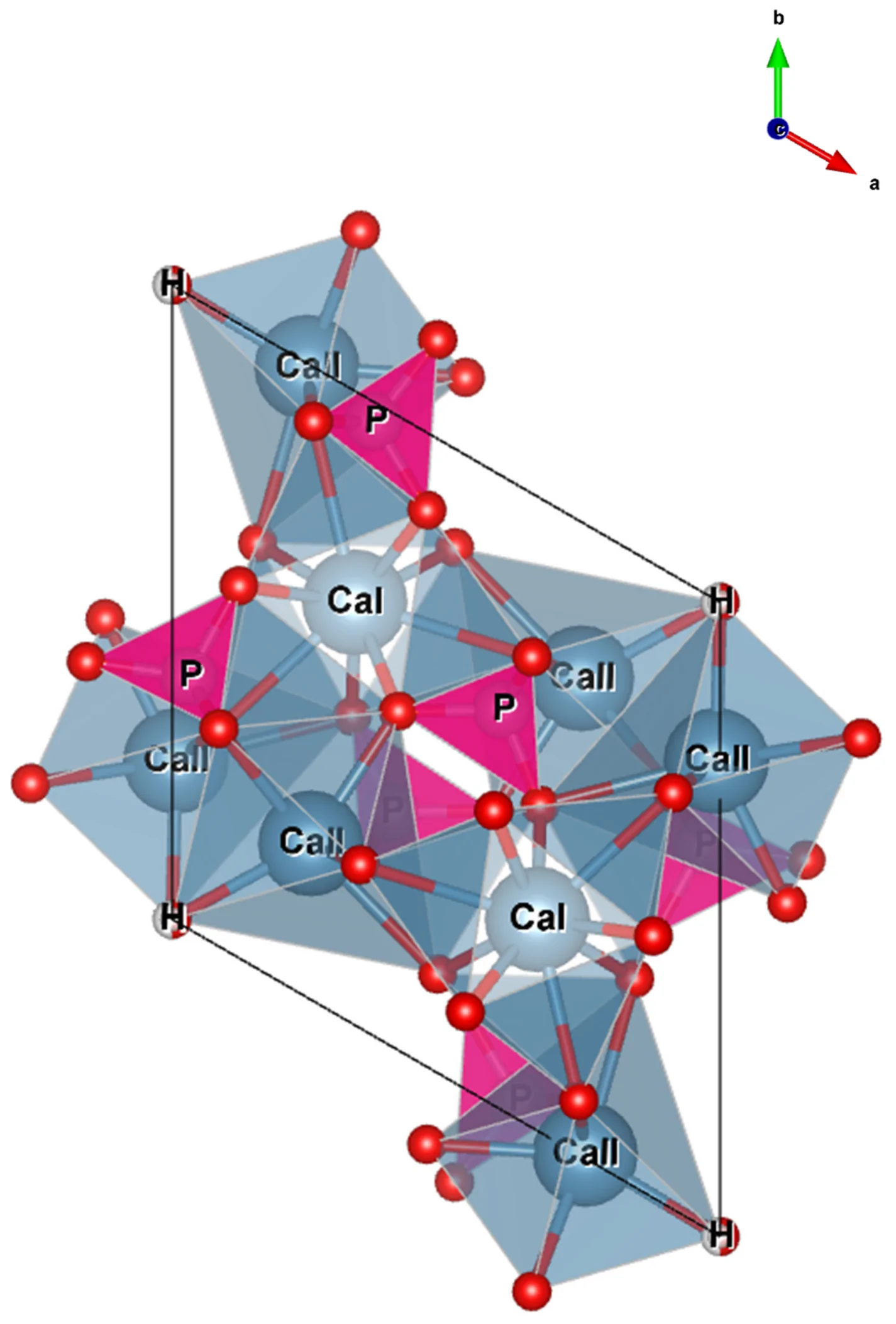
Polyhedral representation of the crystal structure of HA, showing the two crystallographically distinct calcium sites, Ca(I) and Ca(II), the PO_4_^3−^ tetrahedra, and the hydroxyl channels extending along the crystallographic *c*-axis.

The structure of HA, shown in Figure 1, allows partial ionic substitution at different lattice sites. Cationic substitution occurs mainly at Ca^2+^ sites, while anionic substitution may occur at PO_4_^3−^ or OH^−^ sites. Calcium ions in HA occupy two crystallographically distinct positions, known as Ca(I) and Ca(II). The Ca(I) sites are located mainly inside the apatite framework and form column-like arrangements parallel to the crystallographic *c*-axis. These sites are coordinated mainly by oxygen atoms from surrounding PO_4_^3−^ tetrahedra and are relatively regular. In contrast, the Ca(II) sites are positioned around the OH^−^ channels running along the *c*-axis. These sites are more distorted and closer to the channel region, where they interact with hydroxyl groups and phosphate oxygen atoms.

Ionic incorporation into HA is more complex than a simple one-to-one replacement at calcium sites [17]. For divalent dopants, substitution commonly occurs at Ca(I) and/or Ca(II) positions, and the preferred site varies with ionic radius, local coordination, dopant concentration, and synthesis conditions [18,19]. Experimental and computational studies on Sr-substituted HA show that site occupancy is not fixed: mixed substitution can be preferred overall, while Ca(II) occupancy becomes comparably favorable under some wet-chemical, moderate-temperature, and composition-dependent conditions [18,20].

For smaller cations such as Zn^2+^ and Cu^2+^, preference tracks more distorted or lower-coordination calcium environments. In calcium-deficient HA, Zn stabilization is further influenced by vacancy-modified coordination. A first-principles study reported that the defect formation energy reached a minimum at approximately 15 mol% Zn, while the preferred Zn substitution site varied with composition [21]. One crystallographic study suggested a preference for Ca(II) at low Zn content, but an increasing preference for Ca(I) at higher Zn loading, together with appreciable structural water and reduced crystallinity [22]. Additional modeling of Zn/silicate co-substituted HA showed that the lowest-energy Zn configuration can even be an interstitial site near a silicate tetrahedron rather than a regular calcium site, reinforcing that Zn incorporation is chemistry-dependent rather than uniform [17]. For larger divalent ions such as Sr^2+^, accommodation reflects size and thermodynamic context rather than simple radius matching alone [18]. Co-doping studies also confirm that Zn^2+^ and Sr^2+^ replace Ca^2+^ only partially and that the degree of substitution strongly affects hydroxylation, crystallinity, and phase retention [20,23]. By contrast, trivalent and tetravalent dopants generally require explicit charge compensation [19]. Atomistic simulations found that trivalent dopants tend to prefer Ca sites, tetravalent dopants can enter P sites, and compensation through calcium vacancies is generally more favorable than phosphorus-defect schemes [19]. In Ti-modified HA specifically, low Ti contents favor predominant phosphate-site substitution as TiO_4_ units, whereas higher Ti loadings can additionally populate Ca sites depending on synthesis route and concentration [24]. Ti behavior is also preparation-sensitive because excess or thermally treated Ti often segregates into TiO_2_-rich secondary phases, including amorphous Ti-bearing impurities that crystallize as anatase or rutile on calcination [24,25].

**Figure 2:**
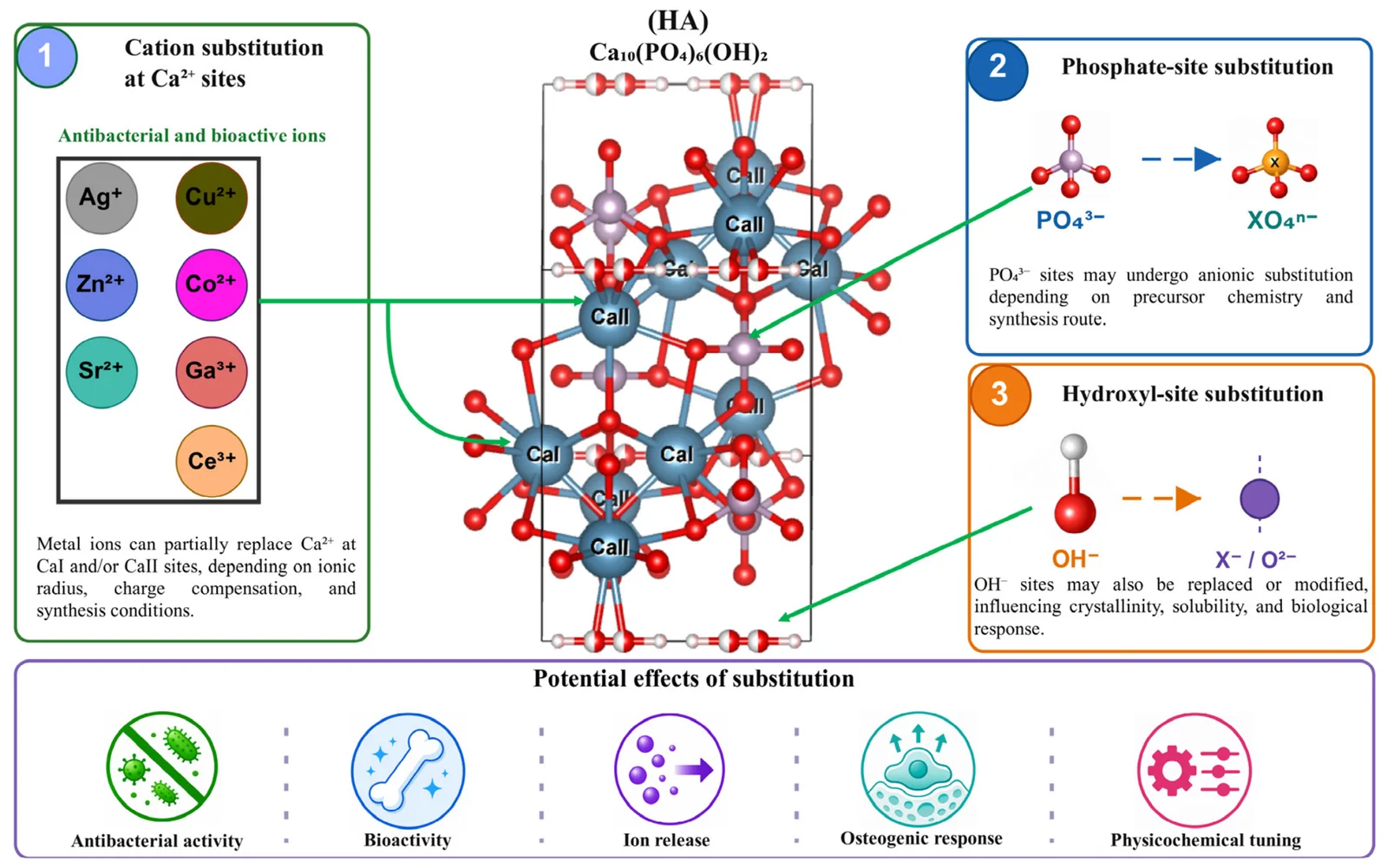
Principal substitution pathways in the HA structure.

As shown in Figure 2, the numerals 1, 2, and 3 denote the three principal classes of substitution discussed in this review: cation substitution at calcium sites, anion substitution at phosphate sites, and anion substitution at hydroxyl-channel sites, respectively. Most metal ions addressed here act primarily through pathway 1, although some compositions indirectly perturb phosphate or hydroxyl environments through charge compensation and local structural distortion.

### 1.3. Clinical Need and Trends in Antibacterial Metal-Substituted Hydroxyapatite

**Figure 3:**
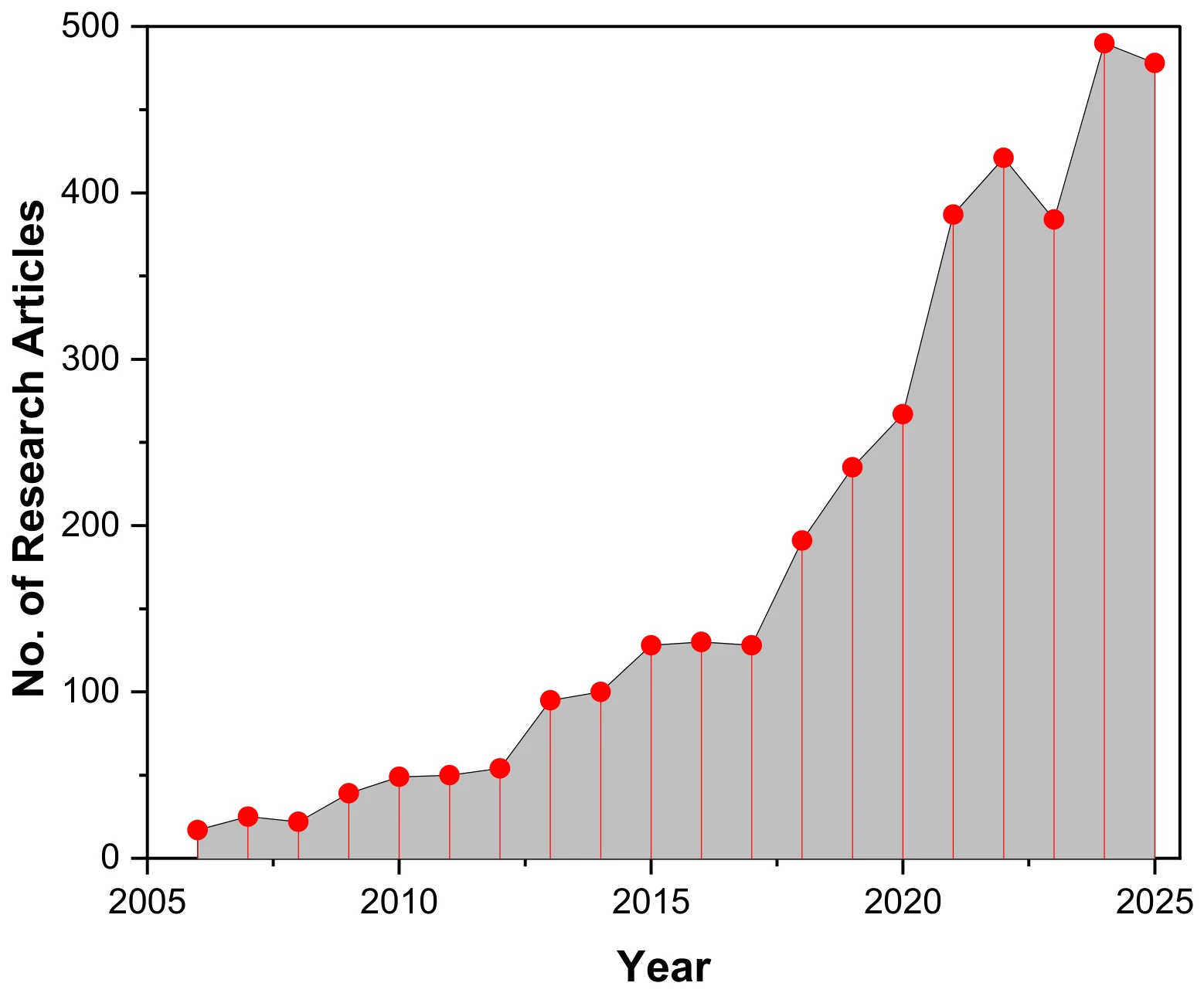
Annual number of research articles on antibacterial hydroxyapatite published in Scopus-indexed journals (2005–2025). Data source: www.scopus.com.

HA-based biomaterials and related CP compounds are commercially available and widely used as bone graft substitutes, implant coatings, and hard-tissue repair materials because of their chemical similarity to the mineral phase of bone and teeth [26,27]. Biomaterial-associated infections remain a major complication in reconstructive, orthopedic, and dental implant surgery, particularly because implanted surfaces can promote bacterial adhesion and biofilm formation [28,29]. After implantation, the material surface interacts with host proteins, cells, and surrounding tissues, while bacterial colonization can disturb the normal host response and reduce the effectiveness of immune defense mechanisms [28,30]. Therefore, systemic antibiotics often require prolonged administration or high local concentrations to control infection, but their effectiveness may be limited by poor penetration into biofilms and infected tissues [28,29]. Implant-related infections are mainly associated with bacterial adhesion, colonization, and biofilm development on the biomaterial surface [28,30]. The number of research articles containing the keywords “antibacterial” + “hydroxyapatite” in Scopus 2005 is summarized in Figure 3. The rapid increase in publications indicates the growing scientific interest in antibacterial metal-substituted HA, driven by the need for biomaterials that combine bioactivity, osteoconductivity, and infection control [31,32].

The substitution of therapeutic metal ions, such as Ag^+^, Cu^2+^, Sr^2+^, Ce^3+^, and Ti^4+^, into HA has become an important strategy in modern biomaterial design because these ions can introduce additional antibacterial, osteogenic, antioxidant, and photocatalytic functions [32,33,34,35]. These ions may act individually by damaging bacterial membranes, disturbing enzyme activity, generating reactive oxygen species (ROS), interfering with deoxyribonucleic acid (DNA) replication, or reducing bacterial adhesion and biofilm formation [32,33,36,37]. Among these dopants, Ag^+^ is one of the most widely studied antimicrobial ions; Ag-doped HA has shown antibacterial activity against both Gram-positive and Gram-negative bacteria, including *Staphylococcus aureus* (*S. aureus*) and *Escherichia coli* (*E. coli*) [33,36]. Cu^2+^-substituted HA has also been reported to enhance antibacterial performance, whereas Sr^2+^ is primarily known for its osteogenic effects but may also contribute to antibacterial activity at optimized concentrations [32,34,38]. Ce-substituted HA has attracted attention because cerium can exist as Ce^3+^ and may provide biological, antioxidant, and antimicrobial functionality depending on its oxidation state and concentration [32,35]. Titanium (Ti)-containing HA or TiO_2_/HA composites are also promising multifunctional systems, particularly because TiO_2_ can generate ROS under light irradiation and provide photoactive antibacterial behavior [37].

Antimicrobial effects should be clearly classified as either bactericidal or bacteriostatic. Bactericidal agents directly kill bacterial cells, whereas bacteriostatic agents suppress bacterial growth without necessarily causing cell death [39]. The final antibacterial response of ion-substituted HA depends strongly on dopant type, ion-release rate, concentration, bacterial strain, exposure time, and cytocompatibility [32,33,36]. Ag-containing CP and bioactive glass systems are particularly attractive because they can release Ag^+^ locally at the implant site; for example, Ag_2_O-doped bioactive glass showed broad-spectrum bactericidal activity against *E. coli*, *Pseudomonas aeruginosa* (*P. aeruginosa*), and *S. aureus* while maintaining bioactive behavior [40]. However, concentrations of Ag^+^ and other therapeutic ions must be carefully optimized because excessive ion release may cause cytotoxicity, reduce crystallinity, disrupt apatite formation, or impair cell compatibility [32,33,40].

HA has been extensively used as an implant, bone-graft, and coating material because its chemical composition resembles the mineral phase of natural hard tissues and because it can support osteoconduction, osseointegration, and strong bonding with bone [14,41]. After implantation, proteins and other biomolecules from physiological fluids are rapidly adsorbed onto the HA surface, forming a conditioning layer that influences subsequent cell attachment, immune response, and bacterial adhesion [42,43]. Although protein adsorption can support favorable host–cell interactions, it may also create binding sites for bacterial attachment if microbial contamination occurs before stable tissue integration [43,44]. Therefore, HA is widely used to repair bone defects and is also applied as a coating on metallic prostheses to improve their biological activity, osteoconductivity, and bone–implant integration [14,45].

Post-surgical orthopedic and dental implant-associated infections remain a major challenge because bacteria can adhere to implant surfaces and develop biofilms that resist both host immune defense and conventional antibiotic therapy [30,44]. These infections are mainly associated with bacterial colonization, biofilm formation, and dysregulated host responses at the implant–tissue interface, rather than only adverse tissue reactions to the implanted material [30,44]. In severe or persistent cases, surgical debridement and even removal or revision of the prosthetic material may be required, thereby increasing patient morbidity, treatment costs, and the risk of further complications [30,46]. Systemic antibiotic therapy is often limited by poor penetration of antibiotics into biofilms and poorly vascularized infected bone tissue, whereas local delivery systems can provide higher drug concentrations at the site of infection while reducing systemic toxicity [45,47]. In this context, porous HA and CP-based materials are promising carriers because their interconnected pores and large surface area can host antibiotics, growth factors, or other therapeutic agents and release them gradually at the target site [47,48]. Several studies have confirmed that HA particles, porous HA scaffolds, and nano-HA systems can be used for local and controlled delivery of antibiotics, anticancer drugs, and other bioactive molecules [47,48].

Owing to the limitations of conventional implant materials and systemic antimicrobial therapy, new strategies are needed to improve the antibacterial performance of HA and HA-like biomaterials while preserving their bioactivity and cytocompatibility [16,30]. One widely investigated approach is the substitution or incorporation of therapeutic metal ions such as Ag^+^, Cu^2+^, Co^2+^, Zn^2+^, Ti^4+^, Ce^3+^, Ga^3+^, and Sr^2+^ into the HA lattice or HA-based coatings [16,32]. These ions may inhibit bacterial adhesion, disrupt bacterial membranes, interfere with enzyme activity or iron metabolism, generate ROS, reduce biofilm formation, and, in some cases, enhance osteogenic activity [16,32,49]. Ag^+^, Cu^2+^, Zn^2+^, and Ga^3+^ are mainly introduced for antibacterial or antibiofilm effects, whereas Sr^2+^ is commonly associated with improved osteogenic response and bone regeneration; therefore, dopant concentration must be optimized to balance antibacterial activity, ion release, and cell compatibility [16,32,49]. Consequently, metal-ion-substituted HA represents a promising multifunctional platform for bone repair applications where both osseointegration and infection control are required [16,32].

### 1.4. Scope of the Review and Broader Biomedical Applications

Although antimicrobial activity and bone regeneration are the principal subjects of this review, metal-substituted HA materials have also been investigated for several complementary biomedical applications, including drug delivery, dental care, implant coatings, and multifunctional biomaterial systems [50,51,52]. The high surface area, porosity, ion-exchange behavior, chemical stability under physiological conditions, and affinity of HA for therapeutic molecules make it a suitable platform for local and controlled drug delivery [51,53,54,55]. Reported applications include antibiotic delivery for bone and implant-associated infections, local treatment approaches for osteomyelitis, delivery of anticancer agents for bone-related malignancies, dental remineralization, photoactive dental materials, and multifunctional surface coatings [37,41,56,57].

Examples discussed in this review include tetracycline-loaded Zn-substituted HA, which combines the antimicrobial contribution of Zn-substituted HA with local antibiotic loading, and doxorubicin-loaded Ce-substituted HA, which shows potential for bone cancer-related drug delivery [58,59]. Metal substitution can influence drug adsorption and release by modifying HA crystallinity, surface charge, dissolution behavior, porosity, surface area, and ion-release characteristics [54,60,61,62]. In Zn-doped HA used as a ciprofloxacin carrier, Zn increased the percentage of drug released while maintaining controlled release, directly demonstrating that dopants can alter delivery kinetics [57]. Nevertheless, the main focus here remains infection-resistant bone and dental biomaterials, especially the balance among antibacterial activity, osteogenic performance, biocompatibility, and tissue integration. At the same time, drug delivery and cancer-related therapy are better treated as complementary applications rather than the central theme [41,51,52].

## 2. Antibacterial Mechanisms of Metal-Substituted Hydroxyapatite

The antibacterial mechanisms of metal-ion-substituted HA are complex and not yet fully understood, as they depend on the dopant ion, oxidation state, ion-release rate, particle size, surface charge, bacterial strain, and exposure conditions [16,32]. However, previous studies suggest that the antibacterial activity of ion-doped HA and related metal-containing biomaterials occurs mainly through three interconnected mechanisms: intracellular disruption, membrane damage, and oxidative stress [16,63,64,65].

First, released metal ions such as Ag^+^, Cu^2+^, Zn^2+^, Co^2+^, and Ga^3+^ may enter bacterial cells through damaged membranes, ion channels, or metal-transport pathways and interfere with essential intracellular processes [58,63,64,65]. After internalization, these ions can bind to proteins, enzymes, thiol groups, nucleic acids, and phosphate-containing biomolecules, leading to the inhibition of respiratory activity, reduced Adenosine Triphosphate (ATP) production, enzyme dysfunction, and disruption of DNA replication or transcription [58,63,64,65]. For example, Ag^+^ can interact with bacterial membranes and intracellular biomolecules, while Zn^2+^ has been reported to interfere with DNA replication and transcription by binding to nucleic acids and proteins [58,65]. Ga^3+^ acts by mimicking Fe^3+^ and disrupting bacterial iron metabolism; because Ga^3+^ cannot undergo normal Fe^3+^/Fe^2+^ redox cycling, it can inhibit iron-dependent enzymes such as ribonucleotide reductase, which is essential for DNA synthesis [66,67].

Second, metal ions can accumulate at the bacterial cell envelope and alter membrane integrity, permeability, and ion transport [63,64,65]. This interaction may damage the cell wall and cytoplasmic membrane, disrupt proton transport and the membrane potential, and ultimately cause leakage of intracellular components [63,64,65]. Ag^+^- and Cu^2+^-containing materials are particularly associated with bacterial membrane damage, impaired respiration, and disruption of cell-envelope function [63,64,65]. These effects are important because the bacterial membrane is responsible for nutrient transport, energy generation, and the maintenance of the proton-motive force required for ATP synthesis [63,64].

**Figure 4:**
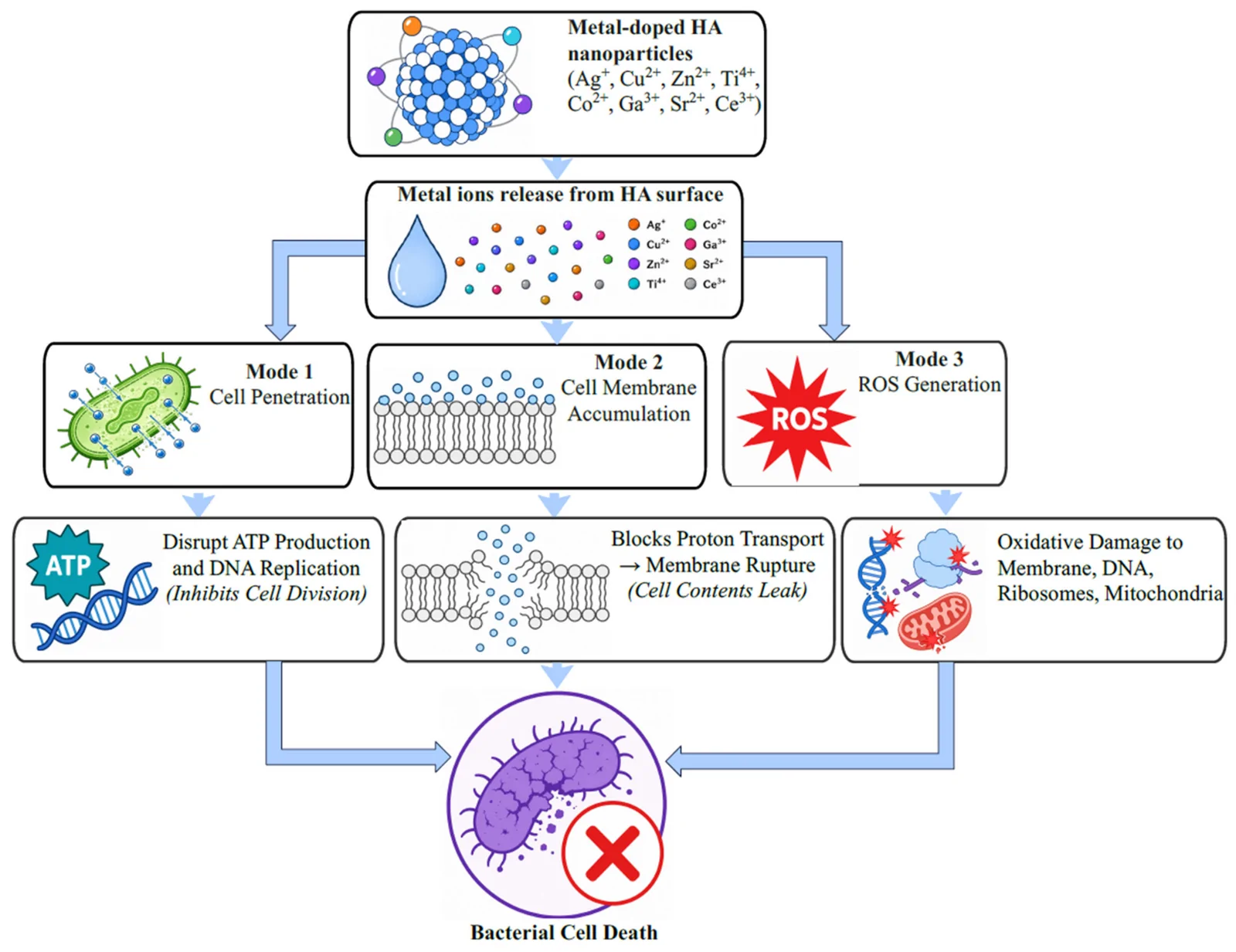
Schematic representation of probable mechanisms involved in metal-doped HA activity.

Third, many metal-containing HA systems can induce oxidative stress by generating or accumulating ROS, such as superoxide radicals, hydroxyl radicals, and hydrogen peroxide [63,64,68]. ROS can oxidize membrane lipids, proteins, enzymes, and DNA, causing irreversible structural and metabolic damage to bacterial cells [63,64,68]. Cu^2+^ ion-containing systems can promote ROS production through redox cycling, while TiO_2_/HA or Ti-containing composites may produce ROS under light irradiation through photocatalytic pathways [64,68]. Cerium-containing HA is more complex because Ce^3+^ redox cycling may show antioxidant or pro-oxidant behavior depending on composition, defect chemistry, and the biological microenvironment [32,69]. The principal antibacterial mechanisms associated with metal-substituted HA are summarized schematically in Figure 4.

Therefore, the antibacterial action of metal-ion-doped HA should not be attributed to a single pathway. Instead, antibacterial performance usually results from the combined effects of ion release, membrane interaction, intracellular metabolic disruption, oxidative stress, and inhibition of biofilm formation [16,32,63,64,65]. Therefore, this review aims to critically summarize recent advances in the design of antibacterial HA-based biomaterials, with particular emphasis on metal-ion substitution strategies involving Ag, Cu, Zn, Ti, Co, Ga, Sr, and Ce. It highlights how synthesis routes, dopant incorporation, phase composition, crystallinity, morphology, ion-release behavior, and surface chemistry influence antibacterial activity, cytocompatibility, osteogenic response, and overall biological performance. In addition, this review discusses the major antibacterial mechanisms of metal-substituted HA systems and identifies key challenges that must be addressed for their safe and effective translation into orthopedic, dental, and implant-related biomedical applications.

### 2.1. Silver-Substituted Hydroxyapatite

Silver (Ag) has long been recognized as one of the most effective inorganic antimicrobial agents and has been widely investigated for biomedical coatings, wound dressings, dental materials, and implant-related applications [65,70]. Ag^+^ and Ag nanoparticles exhibit broad-spectrum antimicrobial activity against Gram-positive and Gram-negative bacteria, fungi, and some viruses; however, their efficacy depends strongly on the form of Ag, particle size, ion-release behavior, microbial strain, exposure time, and testing environment [65,70]. In HA-based biomaterials, Ag can be introduced either as Ag^+^ substituted into the HA lattice, Ag^+^ adsorbed on the surface, Ag nanoparticles embedded in the HA matrix, or secondary Ag-containing phases, depending on the synthesis route and Ag concentration [33,71]. This flexibility makes Ag-containing HA attractive for bone repair and implant coating applications where both osteoconductivity and antibacterial protection are required [33,71,72]. 

The antibacterial mechanism of Ag^+^ is multifactorial and cannot be attributed to a single pathway [65,70]. Released Ag^+^ can interact with negatively charged bacterial membranes, increase membrane permeability, disturb the proton motive force, and cause leakage of intracellular components [65,70]. In addition, Ag^+^ can bind to thiol-containing proteins and enzymes, forming Ag–S interactions that deactivate respiratory enzymes and membrane transport proteins [65,70]. These interactions impair bacterial respiration, reduce ATP production, and disturb essential metabolic processes [65,70]. Ag^+^ can also interact with DNA and other intracellular biomolecules, leading to DNA condensation, inhibition of replication, and disruption of protein synthesis [65,70]. Another important antibacterial pathway involves oxidative stress, in which Ag-containing materials promote the formation of ROS that damage bacterial membranes, proteins, and nucleic acids [65,70].

The antibacterial activity of Ag-containing materials can differ between Gram-positive and Gram-negative bacteria because their cell envelopes are structurally distinct [73]. Gram-negative bacteria have a thin peptidoglycan layer and an outer membrane containing lipopolysaccharide, whereas Gram-positive bacteria lack an outer membrane and possess a much thicker peptidoglycan wall [73,74]. Although some studies report greater Ag activity against Gram-negative species, this trend is not universal, and antibacterial performance also depends on bacterial species, Ag surface chemistry, Ag^+^ release, and experimental conditions [74,75,76].

Ag-substituted HA has attracted considerable attention because it can combine the bioactivity of HA with the antibacterial effect of Ag [33,71,72]. Ciobanu et al. prepared Ag-doped HA nanoparticles using a low-temperature coprecipitation method. They evaluated their antibacterial activity against Gram-positive and Gram-negative species, including *S. aureus*, *Klebsiella pneumoniae* (*K. pneumoniae*), *Providencia stuartii* (*P. stuartii*), *Citrobacter freundii* (*C. freundii*), and *Serratia marcescens* (*S. marcescens*) [72]. Their results showed that antibacterial activity was strongly affected by the Ag content in HA and that Ag-HA nanoparticles exhibited stronger antibacterial activity than pure HA [72]. Similarly, recent studies on nanosized Ag-containing HA confirmed that Ag incorporation can enhance antibacterial and antifungal performance, but also emphasized the need to optimize Ag concentration to avoid cytotoxic effects [33].

The antibacterial efficiency of Ag-HA is controlled by several structural and physicochemical factors, including synthesis method, Ag distribution, crystallinity, particle size, surface area, porosity, and Ag^+^ release rate [33,71,77]. Materials with smaller particle sizes and higher surface areas usually provide greater contact with bacterial cells. They may release Ag^+^ more effectively, thereby improving. However, excessive Ag loading may decrease crystallinity, promote the formation of secondary phases, or increase cytotoxicity, particularly toward osteoblasts, fibroblasts, or mesenchymal stem cells [33,71,78]. Therefore, the antibacterial activity of Ag-HA should always be evaluated together with cytocompatibility, because the same Ag^+^ release that kills bacteria may also impair mammalian cell function at high concentrations [33,78].

Several recent studies confirm this concentration-dependent behavior. Pinchuk et al. reported that Ag-containing nanosized HA showed antibacterial activity against Gram-positive and Gram-negative strains [33]. Lower Ag content being more favorable for osteoblast-related responses is instead supported by other Ag-HA studies showing that increasing Ag improves antibacterial performance but worsens osteoblast adhesion or cytocompatibility [79,80]. Kucukosman et al. showed that Ag-loaded HA prepared by different incorporation methods exhibited distinct antibacterial and cytotoxic responses; embedding Ag nanoparticles in the HA matrix conferred stronger antibacterial performance than simple ionic substitution, while an optimized Ag content provided a better balance between bactericidal activity and cell viability [71]. Sinulingga et al. reported that Ag/Mg- and Ag/Zn-doped HA exhibited strong antibacterial activity against *E. coli* while maintaining higher MC3T3-E1 cell viability than HA with higher Ag loading, suggesting that co-doping can reduce the amount of Ag required while preserving antibacterial efficiency [77]. These findings indicate that Ag-HA design should focus not only on maximizing antibacterial activity but also on achieving controlled Ag^+^ release and acceptable cytocompatibility.

Although Ag-based materials are often described as having a lower tendency to induce resistance than conventional antibiotics due to their multiple antibacterial targets, this statement should be made with caution [81]. Recent reviews have shown that bacteria can develop reduced susceptibility to Ag ions and Ag nanoparticles, or adapt to them, through mechanisms such as efflux pumps, biofilm formation, cell-wall modification, reduction of Ag^+^ to Ag^0^, and stress-response pathways [81]. Therefore, Ag-substituted HA should be used at optimized concentrations and, where possible, combined with other strategies such as co-doping, surface engineering, or controlled release to reduce toxicity and minimize the risk of bacterial adaptation [71,77,81].

Overall, Ag-substituted HA is one of the most promising antibacterial CP systems for bone regeneration and implant-related applications. Its effectiveness arises from the combined action of Ag^+^ release, bacterial membrane disruption, enzyme inhibition, DNA damage, ROS generation, and possible antibiofilm effects [33,65,70,71,72]. However, the clinical success of Ag-HA depends on balancing antibacterial activity with osteogenic performance and mammalian-cell compatibility. For this reason, future studies should systematically evaluate Ag concentration, ion-release kinetics, antibacterial activity against clinically relevant strains, antibiofilm performance, cytocompatibility, and in vivo safety before recommending Ag-HA for routine clinical application [33,71,78].

### 2.2. Copper-Substituted Hydroxyapatite

Copper-substituted HA (Cu-HA) has attracted increasing attention as a multifunctional biomaterial because Cu^2+^ ions can confer antibacterial activity while also promoting angiogenesis and osteogenic responses at controlled concentrations [82,83]. This is particularly important for bone repair and implant coating applications, where bacterial colonization and biofilm formation can compromise osseointegration and lead to implant failure [82,84]. Unlike conventional antibiotic therapy, Cu-containing HA is designed to provide localized antibacterial activity via controlled ion release from the biomaterial surface, thereby reducing bacterial growth near the implant site [83,85].

Cu is an essential trace element in living systems and acts as a cofactor for several enzymes involved in energy metabolism, antioxidant defense, connective tissue formation, and vascular function [82,86]. However, the biological effects of Cu are strongly concentration-dependent; low or moderate concentrations may promote angiogenesis and osteogenesis, whereas excessive Cu release can induce oxidative stress and cytotoxicity in mammalian cells [82,83]. Therefore, the design of Cu-HA requires careful control of Cu content, synthesis route, phase purity, crystallinity, surface area, and ion-release behavior [83,87].

The antibacterial mechanism of Cu-containing biomaterials is generally attributed to the release of Cu^2+^ ions, damage to bacterial membranes, oxidative stress, and intracellular metabolic disruption [68,82]. Released Cu^2+^ ions can interact with negatively charged bacterial cell envelopes, disturb membrane permeability, and damage the cell wall and cytoplasmic membrane [68,82]. After entering the bacterial cell, Cu^2+^ ions can interfere with enzyme activity and disrupt intracellular biochemical processes, leading to damage to proteins and nucleic acids [88,89,90,91]. Cu can also participate in redox reactions that generate ROS, which oxidize membrane lipids, proteins, and nucleic acids, ultimately contributing to bacterial death [82,88,92,93,94,95]. Therefore, the antibacterial action of Cu-HA should be considered a multi-pathway process rather than a single mechanism.

**Figure 5:**
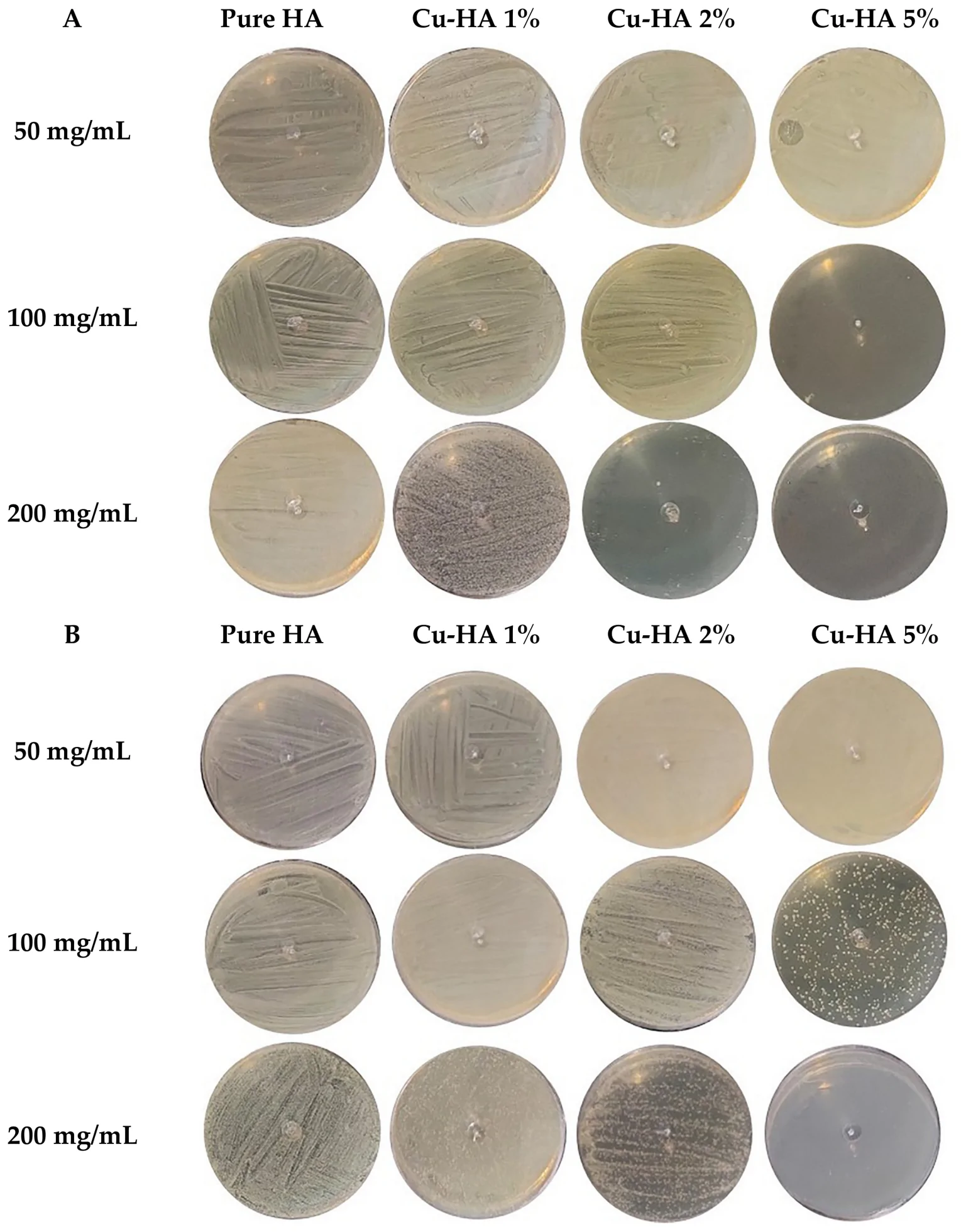
The antibacterial property of powders against *E. coli* (**A**) and *S. aureus* (**B**), after 24 h of cultivation with varied concentrations of Cu-doped HA particles [83]. Reproduced from Noori et al. under the Creative Commons Attribution 4.0 International License (CC BY 4.0).

Several studies have shown that Cu incorporation can modify the crystal structure and physicochemical properties of HA. Noori et al. synthesized Cu-doped HA nanoparticles with 1, 3, and 5% Cu substitution and reported that Cu incorporation slightly decreased lattice parameters and crystallinity, which is consistent with partial replacement of Ca^2+^ by smaller Cu^2+^ ions [83]. Their study also showed that 5% Cu-HA at 200 mg/mL effectively inhibited both *E. coli* and *S. aureus* (Figure 5) while maintaining favorable biocompatibility, osteogenic differentiation, and endothelial cell migration responses [83].

Similarly, Tuntun et al. prepared 0%, 5%, and 10% Cu-doped HA and found that 5% Cu-HA exhibited promising biocompatibility and antimicrobial activity against both Gram-positive and Gram-negative bacteria [85]. These findings indicate that moderate Cu substitution can enhance the biological performance of HA, although the optimum Cu level depends on the synthesis method, particle size, test concentration, and bacterial strain.

The synthesis route plays a major role in determining whether Cu is incorporated into the HA lattice, adsorbed on the surface, or present as a secondary Cu-containing phase [87,96]. Cu-HA has been prepared by wet precipitation, sol–gel, mechanochemical synthesis, spin coating, electrodeposition, plasma spraying, and other coating techniques [85,87,96,97]. Eremina et al. showed that the Cu precursor strongly affects the mechanochemical synthesis of Cu-substituted HA and that Cu^2+^ sources, especially Cu(II) phosphate, are more effective for forming Cu-HA than Cu^0^ or Cu^+^ sources [87]. However, high Cu substitution may reduce the thermal stability of HA and promote decomposition into CuO and Cu-containing phosphate phases during heating [87]. Therefore, phase analysis using X-ray diffraction (XRD), Fourier-transform infrared spectroscopy (FTIR), Raman spectroscopy, X-ray photoelectron spectroscopy (XPS), and elemental mapping are necessary to confirm whether Cu is truly substituted into HA or is present only as a separate phase.

Cu-HA coatings are also promising for metallic implants because they can combine the mechanical strength of Ti or Ti alloys with the bioactivity and antibacterial function of HA [84,97]. Recent studies on Cu-doped HA thin films and coatings have shown improved surface properties, antibacterial activity, and potential biomedical applicability [84,97]. Benali et al. prepared Cu-doped HA coatings and reported that these coatings could be considered potential candidates for antimicrobial biomedical applications [97]. Other studies have also shown that Cu incorporation into CP coatings can alter lattice parameters, increase hardness, and enhance corrosion resistance compared with uncoated Ti surfaces [98]. These results suggest that Cu-HA coatings may be useful for orthopedic and dental implants, provided that Cu release is controlled within a safe biological range.

Despite its advantages, Cu substitution must be carefully optimized because high Cu levels can cause cytotoxicity, reduce crystallinity, alter apatite stability, and affect mammalian cell viability [82,87]. The therapeutic window of Cu is therefore narrower than that of some purely osteogenic ions such as Sr^2+^. For this reason, recent studies increasingly focus on low-dose Cu substitution, Cu-containing coatings, or co-doping strategies with ions such as Sr^2+^, Zn^2+^, Ag^+^, or SeO_3_^2−^ to balance antibacterial activity, angiogenesis, osteogenesis, and cytocompatibility [82,84,99]. Overall, Cu-substituted HA is a promising antibacterial and pro-regenerative biomaterial, but its successful application requires a systematic evaluation of Cu content, ion release, antibacterial activity, biofilm inhibition, cytotoxicity, osteogenic and angiogenic responses, and in vivo safety.

### 2.3. Zinc-Substituted Hydroxyapatite

Zinc (Zn) is an essential trace element involved in many biological processes, including enzyme activity, protein synthesis, DNA synthesis, cell proliferation, immune regulation, wound healing, and skeletal development [100]. In bone metabolism, Zn plays an important role in collagen synthesis, extracellular matrix mineralization, osteoblast differentiation, and bone remodeling [101,102]. Zn-containing biomaterials have therefore attracted considerable interest for bone tissue engineering because Zn can promote osteoblast proliferation and osteogenic differentiation while also suppressing osteoclast differentiation and bone resorption at suitable concentrations [101,103].

In addition to its osteogenic role, Zn^2+^ can provide antibacterial activity, making Zn-substituted HA a promising multifunctional biomaterial for orthopedic, dental, and maxillofacial applications [32,103]. Transition-metal-containing biomaterials, including Zn-, Cu-, and Ag-containing systems, can reduce early bacterial adhesion and biofilm formation on implant surfaces, which is important because early bacterial colonization may compromise osseointegration and lead to implant-associated infection [32,104]. However, the biological response of Zn-containing materials is strongly concentration-dependent; low or moderate Zn levels may enhance cell activity and bone formation, whereas excessive Zn release can induce cytotoxicity, disrupt apatite stability, or adversely affect mammalian cell viability [103,105]. Therefore, the Zn content, synthesis route, crystallinity, particle size, surface area, ion-release behavior, and cytocompatibility must be carefully optimized before Zn-HA can be considered suitable for biomedical use [103,105].

The antibacterial mechanism of Zn-containing HA is usually explained by several combined pathways rather than a single mode of action [32,106]. Released Zn^2+^ ions may interact with negatively charged bacterial cell envelopes, alter membrane permeability, disturb ion transport, and cause leakage of intracellular components [32,106]. Zn^2+^ can also bind to bacterial proteins and enzymes, leading to enzyme inactivation, metabolic inhibition, and disruption of essential cellular processes [32,106]. In addition, Zn-containing materials, especially ZnO-containing or ZnO-associated systems, may generate ROS, which can oxidize membrane lipids, proteins, and nucleic acids and finally reduce bacterial viability [106,107]. Interactions between Zn^2+^ and nucleic acids may also disturb DNA replication and transcription, further contributing to growth inhibition or bacterial death [32,106]. These mechanisms depend strongly on Zn release, material composition, pH, particle size, bacterial strain, and exposure time [32,106,107].

Recent studies have confirmed the antibacterial and biological potential of Zn-HA. Dornelas et al. reported in a 2024 systematic review that Zn incorporation into HA at low concentrations is generally non-cytotoxic and can improve osteoblast-related responses, including cell proliferation, adhesion, osteogenic factor production, and mineralization [103]. Guerra-López et al. synthesized Zn-HA ceramics containing 3, 5, and 10 wt.% Zn^2+^ by wet precipitation and reported antimicrobial activity against *S. aureus*, *Enterococcus faecalis* (*E. faecalis*), *Salmonella typhimurium* (*S. typhimurium*), *E. coli*, and *Candida albicans* (*C. albicans*), with 5 and 10 wt.% Zn-HA showing effective inhibition of microbial growth while maintaining acceptable mesenchymal stem-cell responses [105]. Iconaru et al. developed Zn-doped HA loaded with tetracycline and showed that combining Zn substitution with antibiotic loading can improve antimicrobial performance, indicating that Zn-HA can act as both an antibacterial ceramic and a drug-delivery platform [58]. Similarly, Cuypers et al. reported that Zn-doped HA nanoparticles reduced intracellular survival of *S. aureus* in human THP-1-derived macrophages, supporting the potential of Zn-HA nanoparticles for bone infection-related drug-delivery applications [108].

Zn-HA has also been investigated for the treatment of bone infections and osteomyelitis because conventional systemic antibiotic therapy often shows limited penetration into poorly vascularized infected bone tissue and biofilms [57,108]. In this context, nanocrystalline HA and Zn-HA are attractive because they can mimic bone mineral, provide local ion release, and serve as carriers for antibiotics such as tetracycline, vancomycin, or ciprofloxacin [57,58,108]. Local delivery from Zn-HA-based systems may provide higher antibacterial activity at the site of infection while reducing the need for high systemic antibiotic doses [57,58,108]. Nevertheless, the antibacterial and osteogenic benefits of Zn-HA must be balanced with possible Zn-related cytotoxicity, especially at high concentrations or rapid release rates [103,105]. Overall, Zn-substituted HA represents a promising antibacterial and osteogenic biomaterial, but its practical application requires systematic evaluation of Zn content, release kinetics, antibacterial activity, biofilm inhibition, osteoblast/osteoclast response, cytocompatibility, and in vivo safety.

### 2.4. Titanium-Substituted Hydroxyapatite

Ti-modified HA (Ti–HA) and TiO_2_/HA composites have attracted increasing interest as multifunctional biomaterials for orthopedic, dental, and bone-regeneration applications. This interest is primarily driven by the potential to combine the osteoconductive and bone-bonding properties of HA with the chemical stability, biocompatibility, and photoactive antibacterial behavior of Ti-containing phases [16,24,41]. Unlike Zn and Cu, Ti is not generally considered an essential micronutrient; therefore, its biomedical relevance is mainly associated with Ti implants, TiO_2_ passive layers, and Ti-containing bioactive coatings [16,41]. Pure HA has excellent bioactivity but weak intrinsic antibacterial activity, which has encouraged the development of Ti-modified HA and TiO_2_/HA systems to improve antibacterial performance while maintaining acceptable cytocompatibility [16,24,37,109].

The structural role of Ti in HA is more complex than simple replacement of Ca^2+^ by Ti^4+^. Recent crystal-chemical studies have shown that at low Ti content, Ti^4+^ is primarily incorporated as TiO_4_^4−^ units that replace PO_4_^3−^ groups. In contrast, higher Ti contents may lead to partial occupation of Ca sites and/or formation of amorphous Ti-containing phases that can transform into crystalline TiO_2_ after calcination [24]. Therefore, Ti-containing HA should be described carefully as Ti-substituted HA, Ti-modified HA, or TiO_2_/HA composite depending on the synthesis route, Ti concentration, and structural evidence [24,109]. XRD, FTIR, Raman spectroscopy, XPS, Energy Dispersive X-ray Spectrometer (EDX) mapping, and Rietveld refinement are useful to distinguish true lattice substitution from the formation of secondary TiO_2_-containing phases [24,109].

**Figure 6:**
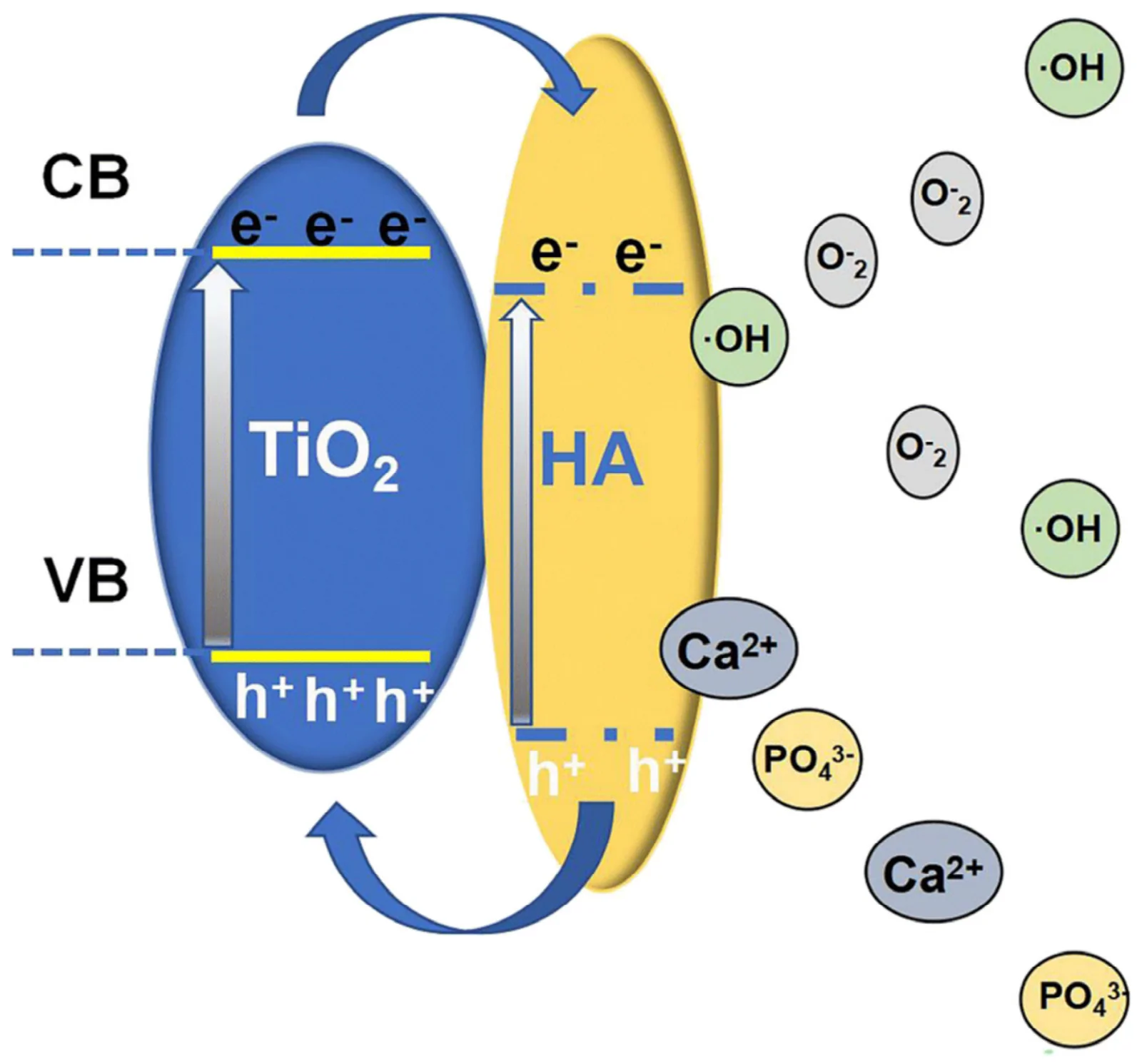
Proposed antibacterial mechanism of TiO_2_/HA nanocomposites. Adapted from Wang et al. (2025) [37] under the Creative Commons Attribution–Non Commercial 3.0 Unported License (CC BY-NC 3.0). The abbreviation “HAP” in the original figure was changed to “HA” for consistency with the terminology used in this review.

The antibacterial activity of Ti-modified HA is mainly attributed to photocatalytic and surface-mediated mechanisms rather than direct Ti^4+^ ion toxicity alone. In TiO_2_/HA composites, light irradiation can generate electron–hole pairs in TiO_2_, which react with oxygen and water to produce ROS, such as hydroxyl radicals, superoxide radicals, and hydrogen peroxide [37,109]. These ROS can damage bacterial cell walls and membranes, oxidize proteins and phospholipids, interfere with DNA, and finally, decrease bacterial viability [37,109,110]. As shown in Figure 6, TiO_2_/HA nanocomposites can exhibit light-assisted antimicrobial activity alongside acceptable cytotoxicity/biocompatibility at optimized concentrations, indicating that antibacterial performance must be evaluated alongside mammalian cell safety [37].

Previous studies have directly investigated the antibacterial and cytotoxicity behavior of Ti-containing HA systems. Li et al. reported that Ti(IV)-substituted HA nanoparticles showed only slight antibacterial activity compared with pure HA at high Ti content, but Ti(IV)-substituted HA was only slightly toxic to osteoblasts. In contrast, Cu-substituted HA showed stronger antibacterial activity but higher cytotoxicity [110]. Fatimah et al. synthesized Ti/HA nanocomposites via a hydrothermal route and reported photoactive antibacterial behavior and cytotoxicity evaluation, thereby supporting Ti/HA as a photoactive antibacterial biomaterial [109]. Korneev et al. further confirmed that Ti modification alters the crystal chemistry and photocatalytic behavior of HA, particularly when Ti-containing phases or HA/TiO_2_ heterostructures form [24]. More recently, Wang et al. synthesized TiO_2_/HA nanocomposites and demonstrated antimicrobial activity, mineralization-promoting behavior, tooth-whitening performance, and good biocompatibility, making this study particularly suitable for illustrating both antibacterial and cytotoxicity-related aspects of TiO_2_/HA systems [37].

Overall, Ti-modified HA and TiO_2_/HA composites are promising antibacterial and bioactive biomaterials, especially for light-assisted antimicrobial treatment, dental applications, and implant coatings. However, their performance strongly depends on Ti content, Ti location within the HA structure, TiO_2_ phase composition, particle size, surface area, crystallinity, light source, irradiation time, bacterial strain, and material dose [24,37,110]. Therefore, future studies should clearly distinguish between true Ti substitution and TiO_2_/HA composite formation, and should systematically evaluate antibacterial activity, cytotoxicity, osteogenic response, photocatalytic ROS generation, ion/particle release, and long-term in vivo safety.

### 2.5. Cobalt-Doped Hydroxyapatite

Cobalt (Co) plays an important biological role as the central metal component of vitamin B_12_ (cobalamin), which is essential for erythrocyte production, DNA synthesis, and normal neurological function. Nevertheless, the effects of free Co^2+^ ions are highly concentration-dependent, and their dosage must therefore be carefully regulated when designing biomaterials [111,112]. In HA, Co^2+^ can partially substitute Ca^2+^ because both ions are divalent, although the smaller ionic radius of Co^2+^ may reduce crystallinity, modify lattice parameters, and affect particle morphology [113,114]. For this reason, Co^2+^-doped HA has been investigated as a multifunctional biomaterial with potential antibacterial, magnetic, angiogenic, osteogenic, and imaging-related functions [113,114,115,116].

The biological interest in Co^2+^-doped HA is mainly related to the ability of Co^2+^ to create a hypoxia-mimicking microenvironment that can stimulate angiogenesis and support bone regeneration at controlled concentrations [115,116]. Kulanthaivel et al. reported that Co^2+^-doped HA promoted proangiogenic and osteogenic responses, suggesting its potential for bone tissue engineering applications [115]. More recently, Yan et al. synthesized Co^2+^-doped HA and evaluated real-time extracts in vitro, confirming that Co^2+^ incorporation can improve bioactivity and cytocompatibility when the dopant level is properly optimized [116]. Safari-Gezaz et al. also showed that Co^2+^-doped HA coatings on Ti-6Al-4V exhibited good corrosion resistance and favorable human osteosarcoma MG-63 cell viability. Among the tested samples, the 10% Co^2+^-doped HA/tetraethyl orthosilicate coating was identified as the most promising composition [117].

Co^2+^-doped HA has also been examined for antimicrobial applications. Tank et al. synthesized Co^2+^-HA nanoparticles using a surfactant-mediated approach and tested it against Gram-negative bacteria, including *P. aeruginosa* and *Shigella flexneri* (*S. flexneri*), and Gram-positive bacteria, including *Micrococcus luteus* (*M. luteus*) and *S. aureus* [113]. Their study reported antimicrobial activity for Co^2+^-HA nanoparticles and also found that the samples were non-hemolytic, suggesting acceptable blood compatibility under the tested conditions [113]. Another hydrothermal Co^2+^-doped HA study reported antibacterial activity against *S. aureus*, along with evaluations of cell adhesion and cell viability, supporting the potential of Co^2+^-doped HA as an antibacterial and bioactive material [114]. Nevertheless, compared with Ag-, Cu-, and Zn-substituted HA, the antibacterial literature on Co^2+^-doped HA remains limited, and the antibacterial mechanism is less clearly established [113,114].

**Figure 7:**
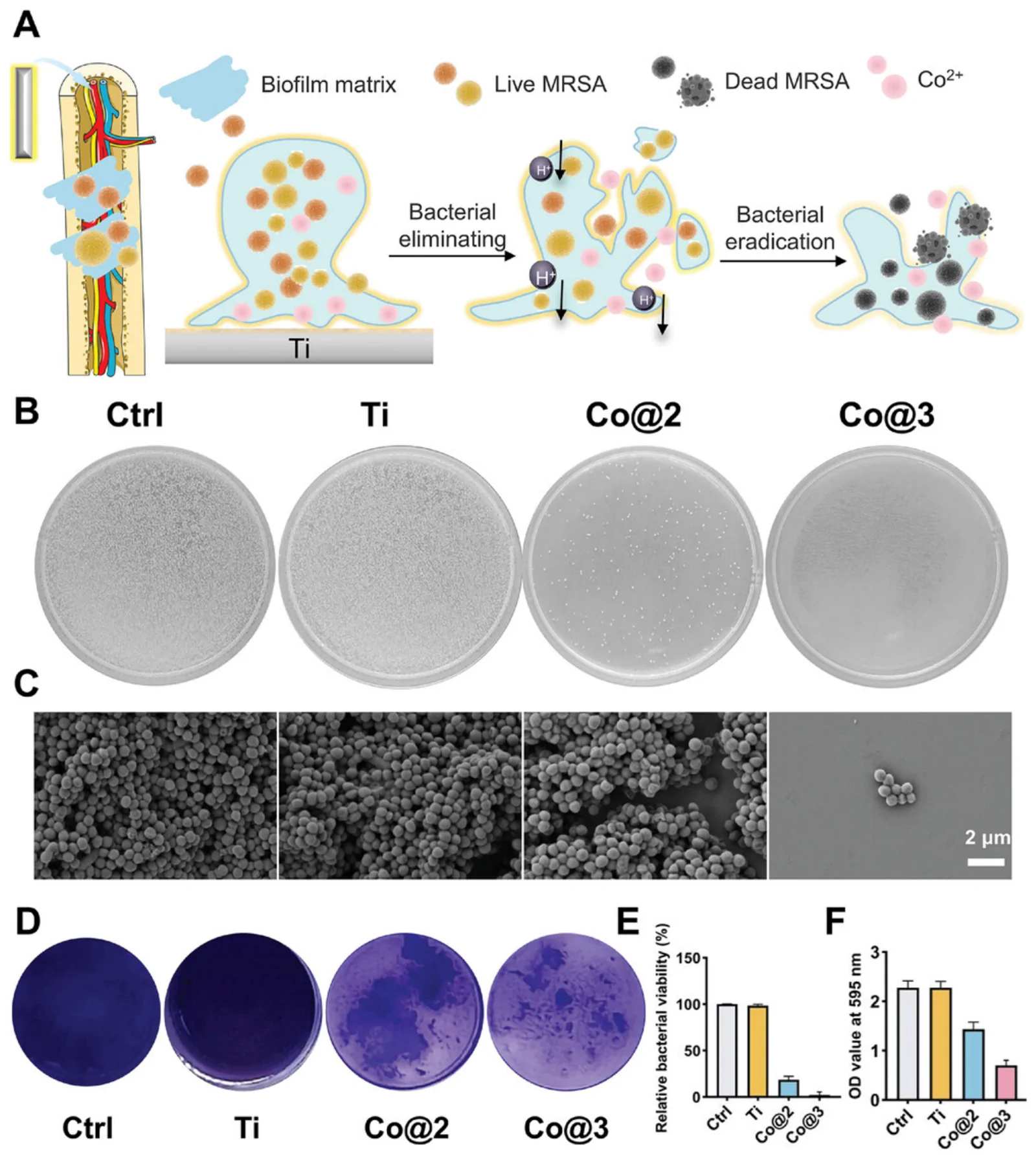
Antibiofilm activity of Co–Ti against MRSA. (**A**) Schematic illustration of the proposed antibiofilm mechanism of Co–Ti, showing disruption of the biofilm matrix followed by bacterial killing and biofilm eradication. (**B**) Representative MRSA colonies grown on agar plates after treatment with the control, Ti, Co@2, and Co@3 samples. (**C**) Scanning Electron Microscope (SEM) images of MRSA biofilms after incubation with the different samples, showing changes in bacterial morphology and biofilm removal. Scale bar: 2 µm. (**D**) Crystal violet staining of biofilm biomass after treatment with the control, Ti, Co@2, and Co@3 samples, indicating reduced biofilm formation, particularly in the Co@3 group. (**E**) Relative viability of MRSA after treatment with the different samples. (**F**) Quantification of biofilm biomass based on optical density at 595 nm (OD595). Data are presented as mean ± standard deviation (SD), *n* = 3 [118]. Reproduced from Yan et al. (2025) under the Creative Commons Attribution 4.0 International License (CC BY 4.0) [118].

The antibacterial activity of Co-containing biomaterials is generally attributed to multiple mechanisms, including Co^2+^ release, interaction with bacterial membranes, disruption of membrane permeability, oxidative stress, enzyme inhibition, and interference with intracellular metabolic pathways [113,118]. Recent work on cobalt-containing implants has shown that controlled Co^2+^ release can disrupt *Methicillin-resistant Staphylococcus aureus* (MRSA) biofilms and simultaneously promote osteointegration through macrophage-mediated immunomodulation; this study provides a useful figure illustrating antibacterial biofilm elimination and the dual antibacterial/osteogenic role of Co-containing implant surfaces (Figure 7) [118]. Although this study is not specifically Co-HA, it is highly relevant for explaining how cobalt-containing biomaterials can combine infection control with bone-regenerative functions [118].

Despite these promising results, Co^2+^-doped HA must be carefully designed, as excessive Co^2+^ release may reduce mammalian cell viability, induce oxidative stress, or trigger inflammatory responses [111,117]. Safari-Gezaz et al. reported that 5% and 10% Co^2+^-containing HA coatings showed favorable MG-63 cell viability, whereas the 20% Co^2+^ coating showed a smaller increase in viability over time, with the 10% Co^2+^ coating exhibiting the best overall performance [117]. Therefore, Co^2+^ concentration, release kinetics, phase purity, crystallinity, particle size, coating stability, antibacterial activity, hemocompatibility, osteogenic response, angiogenic response, and in vivo safety should all be evaluated before Co^2+^-doped HA can be recommended for clinical bone repair applications [113,116,117]. Overall, Co^2+^-doped HA is a promising but less mature antibacterial HA system; its most valuable role may be as a multifunctional material that combines moderate antibacterial activity with angiogenic and osteogenic stimulation at optimized cobalt concentrations.

### 2.6. Gallium-Doped Hydroxyapatite

Gallium-doped HA (Ga-HA) has recently attracted attention as an antibacterial and bioactive biomaterial for bone and dental applications because it combines the osteoconductive nature of HA with the antimicrobial activity of Ga^3+^ ions [66,67,119,120]. Implant-associated infections and antimicrobial resistance remain major challenges in biomaterials science; therefore, non-antibiotic antibacterial strategies based on therapeutic metal ions have become increasingly important [67,119,120]. Compared with highly cytotoxic antimicrobial metals at elevated concentrations, Ga^3+^ is particularly interesting because several studies have reported antibacterial activity alongside acceptable mammalian cell compatibility when the Ga dose and release profile are properly controlled [66,121,122].

Ga is not considered an essential biological element; however, Ga^3+^ has a strong chemical similarity to Fe^3+^ in terms of charge, coordination behavior, and ionic radius [119]. This similarity allows Ga^3+^ to enter bacterial cells through iron-acquisition pathways, especially in iron-dependent pathogens such as *P. aeruginosa* [67,119,120]. Unlike Fe^3+^, Ga^3+^ is redox-inactive under physiological conditions and cannot perform the normal Fe^3+^/Fe^2+^ redox cycling required by many bacterial enzymes [67,119,120]. As a result, Ga^3+^ can disrupt iron- and heme-dependent metabolic pathways, inhibit bacterial respiration, interfere with DNA synthesis, disturb biofilm formation, and reduce bacterial growth [67,119,120]. This “Trojan horse” mechanism makes Ga-based antibacterial systems different from conventional membrane-disrupting metal ions such as Ag^+^ and Cu^2+^ [67,119].

Ga^3+^ is also relevant to bone-related applications because gallium nitrate has historically been investigated for cancer-related hypercalcemia and control of bone resorption [123]. Recent reviews report that gallium nitrate localizes in regions of bone remodeling and can inhibit osteoclast activity, which may explain its hypocalcemic and anti-resorptive effects [123]. Therefore, Ga incorporation into HA is attractive not only for antibacterial activity but also for possible modulation of bone remodeling, provided that the released Ga^3+^ concentration remains within a safe biological range [66,121,123].

Several studies have demonstrated the potential of Ga-containing HA as an antibacterial biomaterial. Kurtjak et al. designed Ga^3+^-containing HA and showed that antibacterial activity against *P. aeruginosa* depended strongly on Ga content, Ga release, particle size, and the composition of the amorphous surface layer [122]. The same study reported that an optimized Ga-HA composition containing approximately 4 wt.% Ga^3+^ exhibited strong antibacterial activity while remaining non-toxic to L929 fibroblasts across the tested concentration range. Mosina et al. later reported that Ga-doped HA exhibited antibacterial activity against *P. aeruginosa* without significantly affecting mammalian cell metabolic activity, supporting Ga-HA as a promising material for controlling bone-related infections [66]. Pajor et al. also prepared HA-based granules containing Ag^+^ or Ga^3+^ and found that Ga-containing HA granules showed antibacterial potential with lower cytotoxicity than Ag-containing analogs under the tested conditions [121].

The antibacterial performance of Ga-HA depends on several physicochemical factors, including Ga concentration, substitution route, crystallinity, surface area, particle size, amorphous surface-layer composition, and Ga^3+^ release kinetics [66,121,122]. Excessive Ga^3+^ release may increase cytotoxicity, while very low release may be insufficient to inhibit bacterial growth [121,122]. Therefore, Ga-HA should be optimized to provide sustained local release of Ga^3+^ at the infected bone site while maintaining cytocompatibility and osteoconductivity [66,121,122]. Overall, Ga-doped HA is a promising antibacterial CP biomaterial, particularly against iron-dependent pathogens such as *P. aeruginosa*; however, further studies are still required to evaluate its antibiofilm activity, cytocompatibility, osteogenic response, ion-release behavior, and in vivo safety prior to clinical translation.

### 2.7. Strontium-Doped Hydroxyapatite

Strontium-doped HA (Sr-HA) has attracted considerable attention as a bioactive CP material for bone repair because Sr^2+^ can partially substitute Ca^2+^ in the HA lattice due to their chemical similarity. The incorporation of Sr^2+^ can modify HA crystallinity, lattice parameters, ion-release behavior, and surface chemistry, thereby influencing its biological performance [124,125,126]. Unlike strongly antibacterial ions such as Ag^+^ or Cu^2+^, Sr^2+^ is mainly recognized for its role in bone regeneration, where it can stimulate osteoblast proliferation and differentiation while reducing osteoclast-mediated bone resorption [124,125]. Therefore, Sr-HA is generally considered more osteogenic and anti-resorptive than primarily antibacterial.

Several recent studies have confirmed that Sr substitution can improve the bioactivity and cytocompatibility of HA-based materials. Qiao et al. synthesized Sr-substituted HA nanoparticles with different Sr/(Ca + Sr) ratios and reported that moderate Sr incorporation enhanced osteoblast proliferation, alkaline phosphatase activity, mineral deposition, and osteogenic gene expression [126]. Similarly, recent reviews on Sr-functionalized biomaterials have emphasized that Sr^2+^ release can support bone remodeling by promoting bone formation and suppressing excessive bone resorption [124,125]. These properties make Sr-HA particularly attractive for bone grafts, dental materials, and implant coatings.

The antibacterial activity of Sr-HA has also been reported, although it is generally less pronounced and more dependent on composition, concentration, particle size, and testing method than the antibacterial activity of Ag-, Cu-, or Zn-doped HA. Jamarun et al. synthesized Sr-doped HA composites using green mussel shells as calcium precursors and evaluated their activity against *S. aureus* and *E. coli*. Their results showed that 5 wt.% Sr-doped HA exhibited antibacterial activity, whereas higher Sr contents of 10 and 20 wt.% did not show the same effect, indicating that Sr concentration must be carefully optimized [34]. Mirković et al. also reported nanocrystalline Sr-doped HA with antimicrobial potential, supporting the idea that nanoscale Sr-HA can contribute to infection-control strategies when properly designed [127]. However, these studies suggest that Sr-HA should not be described as a universally strong antibacterial material; rather, its antibacterial response is composition- and dose-dependent.

The antibacterial mechanism of Sr-containing materials is not yet fully established. Proposed mechanisms include Sr^2+^ release, surface interaction with bacterial membranes, changes in local ionic balance, interference with bacterial metabolism, and possible enhancement of oxidative stress depending on the material system [125,127]. However, in many cases, Sr^2+^ alone provides only moderate antibacterial activity. For this reason, Sr is often combined with more potent antibacterial ions such as Ag^+^, Cu^2+^, Zn^2+^, Ga^3+^, or Ce^3+^ to develop multifunctional HA-based biomaterials with both osteogenic and antibacterial properties [32,128]. For example, Ag/Sr co-substituted HA has been reported to combine the osteogenic benefit of Sr^2+^ with the antibacterial activity of Ag^+^, while Sr/Cu- or Sr/Zn-containing systems are being explored to balance antibacterial performance, osteogenesis, and cytocompatibility [32,128].

Overall, Sr-doped HA is a promising biomaterial for bone regeneration because of its strong osteogenic, anti-resorptive, and immunomodulatory effects [129,130,131,132]. Its antibacterial activity is promising but should be interpreted with caution, as it varies with Sr concentration, synthesis or coating method, surface characteristics, ion-release behavior, bacterial strain, and assay conditions [127,133,134,135]. Future work should integrate release-kinetics analysis, antibacterial and antibiofilm testing, osteoblast and osteoclast response, cytocompatibility, coating stability, and in vivo bone-repair evaluation to support clinical translation of Sr-HA-based bone-repair systems [130,131,136,137,138].

### 2.8. Cerium-Doped Hydroxyapatite

Cerium-doped HA (Ce-HA) has attracted increasing attention as a multifunctional CP biomaterial because it combines the osteoconductive nature of HA with the redox-related biological activity of cerium species [16,32,139]. Cerium is a lanthanide element and is not considered essential in mammalian physiology; however, Ce-containing compounds and cerium oxide nanoparticles have been widely investigated for biomedical applications due to their antibacterial, antioxidant, anti-inflammatory, and tissue-regenerative properties [16,139,140]. Cerium’s biological activity is mainly associated with its ability to switch between Ce^3+^ and Ce^4+^ oxidation states, together with oxygen-vacancy-mediated redox behavior, which enables CeO_2_ nanomaterials to mimic antioxidant enzymes and regulate ROS through their surface Ce^3+^/Ce^4+^ ratio [139,140].

In HA, Ce^3+^ ions can be incorporated mainly through substitution at Ca^2+^ sites because their ionic radius is reasonably compatible with that of Ca^2+^, although charge compensation and local lattice distortion are required [16,69]. Recent nanoscale structural studies suggest that Ce^3+^ can replace Ca^2+^ at the two nonequivalent calcium sites in HA, while heat treatment can modify the crystallinity and defect structure of Ce-HA [69]. Earlier XRD studies also showed that increasing the Ce content can expand the HA lattice parameters and reduce the crystallite size, confirming that cerium incorporation alters the crystal chemistry and morphology of HA [141]. These structural changes may influence surface charge, ion release, protein adsorption, and microbial interaction, which are important for antibacterial and biological performance [16,32,141].

The antibacterial behavior of Ce-HA remains debated and appears to be strongly dependent on Ce concentration, oxidation state, synthesis method, crystallinity, surface area, and bacterial strain [16,32,142]. Proposed antibacterial mechanisms include interactions between Ce ions or Ce-containing surfaces and bacterial cell envelopes, disruption of membrane integrity, modulation of oxidative stress, enzyme inhibition, and interference with bacterial adhesion or biofilm formation [139,140,142]. Cerium oxide systems can exhibit either antioxidant or pro-oxidant behavior depending on pH, the reversibility of Ce^3+^/Ce^4+^ redox cycling, and the formation and concentration of oxygen vacancies, which together make their biological effects strongly context-dependent [139,140]. Therefore, Ce-HA should not be described as universally antibacterial; rather, its antibacterial activity must be evaluated for each composition and test condition.

Several studies have reported promising antibacterial performance for Ce-substituted HA. Ciobanu et al. prepared Ce-doped HA nanoparticles by coprecipitation and reported that Ce-HA with xCe = 0.03 and 0.05 showed significant antibacterial activity against *S. aureus* and *E. coli* compared with pure HA [141]. Predoi et al. developed Ce-doped HA coatings via radio-frequency (RF) magnetron sputtering and reported antimicrobial activity in both Ce-HA suspensions and Ce-HA-coated surfaces, supporting the use of Ce-HA as a coating material for biomedical applications [142]. Singh and Jain synthesized Ce-substituted HA nanoparticles and incorporated them into an orthodontic adhesive; the Ce-HA adhesive showed concentration-dependent antibacterial activity against *S. aureus*, *Lactobacillus acidophilus* (*L. acidophilus*), and *Streptococcus mutans* (*S. mutans*), indicating potential use in dental materials [35]. Moaness et al. also prepared Ce-substituted HA nanoparticles with 3–7 wt.% Ce and reported antimicrobial/antifungal activity, simulated body fluid (SBF) bioactivity, good MG-63 cytocompatibility, and doxorubicin delivery potential for bone cancer-related applications [59].

However, recent evidence also shows that Ce substitution does not always produce strong antibacterial activity. Ivanković et al. compared Ce-, Cu-, Ga-, Mn-, and Ag-substituted HA against clinically relevant multidrug-resistant bacteria and found that all materials except Cu-substituted HA were non-cytotoxic. However, Ce-substituted HA showed no clear antibacterial effect under their testing conditions [143]. This finding is important because it shows that Ce-HA antibacterial performance is not universal and may depend on the testing protocol, bacterial strain, Ce release, surface chemistry, and whether the material is used as a powder, suspension, coating, or composite [143]. Therefore, future studies should combine antibacterial assays with cytocompatibility, ion-release analysis, ROS measurements, biofilm tests, and surface characterization to better explain the biological performance of Ce-HA.

Overall, Ce-doped HA is a promising multifunctional material for bone and dental applications because it may combine HA bioactivity with cerium-related antioxidant, antibacterial, and regenerative effects [16,32,139,140]. Nevertheless, its antibacterial potential should be discussed cautiously because published results are sometimes contradictory. The most realistic conclusion is that optimized Ce content, controlled Ce^3+^/Ce^4+^ ratio, suitable synthesis method, and appropriate surface chemistry are required to achieve useful antibacterial activity while preserving cytocompatibility and osteoconductivity.

Numerous studies have demonstrated that the HA lattice can accommodate a wide range of ionic substitutions at Ca^2+^, PO_4_^3−^, and OH^−^ sites, allowing its physicochemical and biological properties to be tailored for biomedical applications [16,31,32]. These substitutions may involve monovalent, divalent, trivalent, or tetravalent ions, although their successful incorporation depends on ionic radius, oxidation state, coordination environment, dopant concentration, synthesis route, and charge-compensation mechanism [16,31,32,144]. In antibacterial HA-based biomaterials, ions such as Ag^+^, Cu^2+^, Zn^2+^, Co^2+^, Ga^3+^, Sr^2+^, Ce^3+^, and Ti^4+^ have been investigated because they can reduce bacterial adhesion, disturb bacterial membranes, interfere with intracellular metabolism, generate or regulate ROS, and inhibit biofilm formation [14,31,32,144,145]. However, the antibacterial efficacy of each dopant is strongly dependent on dopant concentration, release behavior, particle size, crystallinity, surface area, bacterial strain, and culture conditions [14,144,145,146]. Therefore, Table 1 summarizes the reported antibacterial responses of different metal-doped HA systems against selected pathogenic bacteria, along with the minimum effective dopant concentrations reported in the literature.

It should be noted that the reported minimum effective dose values cannot be directly compared among all studies because the units, synthesis methods, sample forms, bacterial concentrations, culture times, and antibacterial assays differ significantly. Therefore, the table should be interpreted as a summary of reported positive responses in the literature rather than a direct ranking of dopant efficiency.

## 3. Biological Safety and Regenerative Mechanisms

The regenerative activity of metal-substituted HA involves interconnected molecular, cellular, and tissue-level processes [154,155,156]. Following implantation, proteins are adsorbed onto the HA surface, and this adsorption state helps regulate cell adhesion and subsequent tissue responses [157,158,159]. HA surface chemistry, hydration state, and crystallinity shape the amount and conformation of adsorbed proteins, which then influence integrin signaling, inflammatory behavior, and cell attachment [155,157,160].

The release of calcium, phosphate, and therapeutic metal ions from HA-based systems can promote mesenchymal stem cell and osteoblast differentiation, extracellular matrix production, and mineralization [161,162]. Calcium and phosphate release is directly linked to biomineralization and osteogenic differentiation in HA-containing constructs [161,163,164]. The surrounding Ca/P release ratio also affects the osteoinductivity and osteoconductivity of CP bioceramics, including HA [165]. Selected ions provide additional functions. Sr and Zn support osteogenic activity and can suppress osteoclast-mediated resorption [166,167,168,169]. Sr-containing HA systems promoted osteoblast viability and activity while inhibiting osteoclastogenesis in co-culture models [166,170]. Zn/Sr co-substituted HA also enhanced cell attachment, proliferation, matrix mineralization, and osteogenic marker expression compared with pristine or single-substituted HA [156,169].

Controlled Cu^2+^ and Co^2+^ release can enhance angiogenic signaling and vascularized bone formation, but these effects are strongly dose-dependent: sustained or tuned release promotes Vascular endothelial growth factor (VEGF)/Hypoxia-Inducible Factor 1-alpha (HIF-1α)-associated endothelial and bone-regenerative responses, whereas excessive concentrations can reduce cell viability or blunt angiogenic network formation [171,172,173,174,175]. Cu-containing HA systems promoted endothelial migration and angiogenesis, while also supporting osteoblast proliferation and mineralization in optimized formulations [176]. Co ions containing apatitic materials increased VEGF and von Willebrand factor expression, although cobalt also reduced metabolic activity in one Human mesenchymal stem cell (hMSC) study, indicating a narrower therapeutic window than Sr [170]. HA-based materials can also influence macrophage activity and the local immune environment, thereby affecting inflammation, osseointegration, and bone remodeling [154,160,168]. Sr-containing or Zn/Sr-containing HA systems promoted a more pro-regenerative immune microenvironment through macrophage modulation [154,160].

HA offers important advantages over many polymeric or metallic nanomaterials because it is chemically similar to bone mineral, osteoconductive, and capable of forming a direct mineralized interface with surrounding tissue [156,163,167]. HA is widely valued as a bone-facing phase in composites because the polymer provides structural support, while HA promotes cell proliferation, osteoinduction, and mineralized tissue integration [163]. Metal-substituted HA is not necessarily superior to all other nanostructured materials, because regenerative performance depends strongly on morphology, crystallinity, porosity, dopant concentration, ion-release behavior, and biological context [154,155]. Ionic substitution in HA changes lattice parameters, crystallinity, morphology, surface characteristics, solubility, and bioactivity, which helps explain why biological outcome is governed not only by composition but also by the structural and interfacial changes that substitution introduces [177,178,179,180,181]. In one recent Cu/Sr-HA study, moderate crystallinity was optimal, while different HA loading and pore compression were identified as variables likely to alter degradation, cell compatibility, and infiltration [155]. Its main advantage is therefore the combination of a bone-like mineral matrix with bioactive ion delivery, including regenerative and sometimes antibacterial functions [155,156].

Biological safety must be evaluated alongside antibacterial and regenerative performance, as the same ions that stimulate osteogenesis, angiogenesis, or antimicrobial activity can become cytotoxic outside the optimal dose range [155,168]. Several optimized metal-substituted HA systems showed acceptable compatibility with osteoblast-like cells, mesenchymal stem cells, endothelial cells, and fibroblasts under controlled conditions [157,162].

Examples from this set include co-substituted HA cements that were non-cytotoxic in fibroblasts, endothelial cells, and osteoblasts while improving osteoblast adhesion and proliferation [162]. Zn/Sr-substituted HA nanoparticles and Cu-HA-containing printed scaffolds were also reported to be highly biocompatible and to enhance osteogenic outcomes [155,169].

## 4. Commercial and Translational Examples

Commercial translation of metal-substitute HA remains more limited than the large number of experimental studies might suggest. Most commercially available HA materials are conventional implant coatings or bone-graft materials, whereas Ag-containing HA coatings mainly represent true metal-functionalized HA products. Representative examples are summarized in Table 2.

These examples demonstrate the translational potential of ion-functionalized HA; however, most Cu-, Zn-, Co-, Ga-, Sr-, Ce-, and Ti-modified HA systems remain at the experimental or preclinical stage.

## 5. Conclusions and Future Perspectives

Metal-ion substitution is a versatile strategy for transforming HA from a primarily osteoconductive CP into a multifunctional biomaterial capable of supporting tissue regeneration and controlling infection. Its biological performance depends not only on the type and concentration of the dopant but also on lattice occupancy, charge compensation, phase composition, crystallinity, morphology, surface properties, material form, and ion-release kinetics.

Ag-, Cu-, Zn-, and Ga-containing HA generally show the most consistent antibacterial effects. Ag provides broad-spectrum activity but may become cytotoxic at excessive concentrations. Cu and Zn combine antibacterial activity with osteogenic and, in the case of Cu, angiogenic effects, although careful dose control is required. Ga is particularly promising against iron-dependent pathogens because it disrupts bacterial iron metabolism. Ti-, Co-, Sr-, and Ce-containing systems show more condition-dependent behavior. TiO_2_/HA is primarily suited to photoactivated antimicrobial applications; Co may support angiogenesis and osteogenesis; Sr is primarily osteogenic and anti-resorptive; and Ce may provide antioxidant, regenerative, and antibacterial functions depending on its concentration and oxidation state.

The principal challenge is to balance antibacterial and antibiofilm activity with cytocompatibility, osteogenesis, angiogenesis, immune regulation, and material stability. Ag-containing HA coatings currently dominate commercial translation, whereas most other metal-modified HA systems remain at the experimental or pre-clinical stage. Nevertheless, these materials show considerable potential for infection-resistant bone grafts, implant coatings, scaffolds, and local drug-delivery systems.

Future research should prioritize standardized antibacterial and biofilm-testing protocols, accurate confirmation of lattice substitution, and systematic evaluation of ion-release kinetics. Biological testing should extend beyond planktonic bacteria and inhibition-zone assays to clinically relevant mono- and polymicrobial biofilms formed on realistic implant surfaces. Antibacterial studies should also be integrated with cytotoxicity, hemocompatibility, osteogenic, angiogenic, and immunomodulatory assessments.

Rational co-substitutions, such as Ag/Sr, Cu/Sr, Zn/Sr, and Ag/Zn, may reduce the required dose of potentially toxic antibacterial ions while preserving regenerative activity. Clinical translation will further require reproducible large-scale manufacturing, effective sterilization, coating-stability testing, and long-term in vivo studies evaluating infection control, osseointegration, systemic metal accumulation, and organ safety.

## Figures and Tables

**Table 1:** Recent studies on antibacterial metal-ion-substituted HA-based biomaterials.

Ion	HA-Based Material	Preparation/Material Form	Metal Ion Amount (How Reported)	Tested Bacteria	Main Reported Result	Ref.
Ag^+^	Ag-substituted HA powder	Wet coprecipitation; powder	Ag: 0.54 wt.% (nominal)	*S. aureus*, *Staphylococcus epidermidis* (*S. epidermidis*), *E. coli*, *P. aeruginosa*	Produced inhibition zones of 12, 13, 11, and 12 mm against *S. aureus*, *S. epidermidis*, *E. coli*, and *P. aeruginosa*, respectively.	[121]
Ag^+^	Ag-containing alginate/HA granules	Alginate crosslinking using Ag-HA powder; granules	1 g Ag-HA dispersed in 10 mL alginate suspension	*S. aureus*, *S. epidermidis*, *E. coli*, *P. aeruginosa*	Produced inhibition zones of 10, 10, 8, and 10 mm against *S. aureus*, *S. epidermidis*, *E. coli*, and *P. aeruginosa*, respectively.	[121]
Ag^+^	Ag-HA powder suspension	Wet synthesis; powder suspension	Ag-HA suspension: 0.1 and 1 mg/mL	*S. aureus*, *S. epidermidis*, *E. coli*, *P. aeruginosa*	At 1 mg/mL, Ag-HA completely or nearly completely reduced the viability of all tested strains.	[121]
Ag^+^	Ag-doped CP coating on Ti pins	Electrospray deposition of Ag-doped HA/CP powder on Ti pins, followed by RF sintering; coating	Not reported	*S. epidermidis* clinical isolate	Reduced bacterial colonization on Ti pins compared with both the undoped coating and uncoated Ti.	[147]
Ag^+^	Ag-doped HA layer	Laser deposition; coating	Ag: 0.06 and 14 at. %	*E. coli*, *Bacillus subtilis* (*B. subtilis*)	Antibacterial efficacy ranged from 4% to 100%, depending on Ag content and test conditions.	[148]
Ag^+^	Ag-HA/functionalized multiwall carbon nanotube (HA/f-MWCNT) composite film	Spray pyrolysis on 316L stainless steel; composite coating	Ag: 1, 3, and 5 wt.%	*B. subtilis*, *S. aureus*, *S. flexneri*, *E. coli*	Antibacterial activity increased with increasing Ag content; the minimum inhibitory concentration decreased from 0.25 to 0.125 mg.	[149]
Ag^+^/Sr^2+^	Ag/Sr co-doped HA coating on Ti	Hydrothermal deposition on Ti; coating	Ag: ~1 mol%; Sr: ~10 mol%; co-doped coating: ~1 mol% Ag + ~10 mol% Sr	*S. aureus*, *E. coli*	Ag-containing coatings showed strong antibacterial activity, whereas Ti, HA, and 10Sr showed no detectable activity. Bacterial counts decreased by ~100% and 99% against *E. coli* and by ~96% and 95% against *S. aureus* for Ag0.1 and Sr/Ag, respectively.	[150]
Cu^2+^	Cu-doped HA nanoparticles	Wet chemical synthesis; nanoparticles	Cu: 1, 3, and 5%	*E. coli*, *S. aureus*	At 200 mg/mL, 5% Cu-HA nearly eradicated *S. aureus* and completely inhibited *E. coli*; at the same concentration, 2% Cu-HA also completely inhibited *E. coli*.	[83]
Cu^2+^	Cu-doped HA powder	Wet synthesis; powder	Cu: 0, 5, and 10%	*E. coli*, *S. aureus*	The 5% Cu-HA composition showed the best antimicrobial performance while retaining good hemocompatibility and non-cytotoxicity. The 10% Cu-HA sample remained biocompatible but showed lower antimicrobial activity and slightly reduced hemocompatibility.	[85]
Cu^2+^	Cu-doped HA coating	Powder synthesis followed by vacuum deposition; coating	Ca_10−x_Cu_x_(PO_4_)_6_(OH)_2_, x = 0.03	*P. aeruginosa*	Reduced *P. aeruginosa* attachment and biofilm formation after 24 h, with a further reduction after 72 h, while maintaining good compatibility with HeLa and MG-63 cells.	[97]
Cu^2+^/Sr^2+^	Sr/Cu co-doped β-TCP	Solid-state synthesis; powder	Cu: 9.5 mol% (constant); Sr: 0–42 mol% in Ca_9.5−x_Sr_x_Cu(PO_4_)_7_	*E. coli*, *S. aureus*	Showed significant antibacterial activity against both strains, concentration-dependent cytotoxicity, and improved cell proliferation and osteogenesis at lower concentrations. Compositions with x ≤ 3 were proposed as candidate bone substitutes.	[38]
Se^4+^ and/or Cu^2+^	Se- and/or Cu-substituted HA	Aqueous precipitation followed by freeze-drying; powder	Cu: 5 mol% as Cu/(Ca + Cu); Se: 1.96 mol% as Se/(P + Se)	*S. mutans*, *C. albicans*	The co-substituted SeCuHA sample showed the strongest antimicrobial activity, with inhibition zones of 25 ± 0.6 mm against *S. mutans* and 32 ± 0.41 mm against *C. albicans*; activity against *S. mutans* exceeded that of the positive control (19 ± 0.28 mm).	[99]
Zn^2+^	Zn-doped HA ceramic	Wet precipitation followed by sintering at 700 or 800 °C; dense ceramic	Zn: 3, 5, and 10 wt.%	*S. aureus*, *E. faecalis*, *P. aeruginosa*, *E. coli*, *C. albicans*	Zn-doped HA, particularly 10 wt.% Zn-HA showed broad antimicrobial activity while preserving stem-cell viability; lower Zn contents produced the clearest osteogenic improvements.	[105]
Zn^2+^	Tetracycline-enriched Zn-doped HA powder	Adapted coprecipitation; powder	Zn: 10 mol% as Zn/(Ca + Zn)	*S. aureus*, *E. coli*, C. *albicans*	Both ZnHA and ZnHATe were antimicrobial, but tetracycline loading enhanced the effect, producing bactericidal activity against *S. aureus*, stronger bacteriostatic activity against *E. coli*, and improved antifungal activity against *C. albicans*. Both materials remained biocompatible with hFOB 1.19 cells.	[58]
Zn^2+^	Zn-doped HA nanoparticles combined with vancomycin-loaded gelatin nanoparticles	ZnHA nanoparticles combined with vancomycin-loaded gelatin nanoparticles	Zn: 0, 10, 15, and 20 mol%	Intracellular *S. aureus* in THP-1-derived macrophages	Both ZnHA and vancomycin-loaded gelatin nanoparticles reduced intracellular *S. aureus* survival, showed >75% lysosomal colocalization after macrophage uptake, and did not reduce THP-1 cell viability.	[108]
Ti^4+^	TiHA nanocomposite	Ti/HA nanocomposite synthesized by coprecipitation followed by hydrothermal treatment at 150 °C	Ti content reported as 5, 10, and 20 wt.%	*E. coli*, *S. aureus*, *K. pneumoniae*, *Streptococcus pyogenes* (*S. pyogenes*)	Ti/HA showed photocatalytic and antibacterial activity; 20 wt.% Ti/HA gave the highest methyl violet degradation (96.46%) and MICs of 25 μg/mL against *E. coli* and *S. aureus*, and 50 μg/mL against *K. pneumoniae* and *S. pyogenes.*	[109]
TiO_2_	TiO_2_/HA nanocomposite	Hydrothermal synthesis; nanocomposite	TiO_2_: Ca molar ratios of 2:1, 1:1, and 1:2	*S. mutans*	The 1:2 TiO_2_:HA composition showed the strongest antibacterial effect. TiO_2_/HA combined with LED irradiation significantly reduced colony formation; in vivo, it removed plaque and prevented visible bacterial accumulation in rats.	[37]
Co^2+^	Co-substituted silicate-containing HA powder	Microwave-assisted wet precipitation; powder	Co substitution for Ca: x = 0, 0.2, 0.6, and 1.0	*P. aeruginosa*, *S. aureus*	Antibacterial activity increased with increasing Co^2+^ content. HA, SiHA, and the lowest-Co composition were inactive under the reported conditions.	[151]
Co^2+^	Co-substituted HA powder	Wet chemical synthesis; powder	Co substitution at Ca sites or OH channels: x = 0, 0.2, and 0.3	*E. coli*, *S. aureus*	Undoped HA showed no bactericidal effect, whereas Co-substituted HA inhibited both strains. Activity increased with Co content and was strongest when Co occupied Ca sites, consistent with greater Co release.	[152]
Ga^3+^	Ga-doped HA	Wet chemical precipitation	Target Ga: 2, 4, 6.3, and 8 wt.%; measured Ga: up to 6.9 ± 0.5 wt.%	*P. aeruginosa*, *E. coli*, *S. aureus*, *S. epidermidis*, *and S. pyogenes*	Antibacterial activity increased with Ga incorporation. Samples containing 5.5 ± 0.1 and 6.9 ± 0.5 wt.% Ga inhibited all five strains, with the greatest effect against *P. aeruginosa*.	[66]
Ga^3+^	Ga-, Mg-, and carbonate-co-doped HA nanophase	Low-temperature aqueous precipitation; nanopowder	Ga/Ca = 0.025, 0.05, or 0.1; co-doped samples: Ga/Ca = 0.025 or 0.05, Mg/Ca = 0.1, and CO_3_/PO_4_ = 0.1	*E. coli*, *P. aeruginosa*, *S. aureus*, *C. albicans*	Ga doping produced antibacterial activity, and combined Ga/Mg/carbonate doping further enhanced the effect. Undoped HA consistently showed the lowest antimicrobial performance.	[153]
Sr^2+^	Sr-doped HA derived from green mussel shells	Sol–gel synthesis, drying at 110 °C, and calcination at 600 °C; powder	Sr: 5, 10, and 20 wt.%	*S. aureus*, *E. coli*	Only the 5 wt.% Sr-HA sample showed an inhibition zone; undoped HA and the 10 and 20 wt.% Sr-HA samples showed no detectable inhibition.	[34]
Sr^2+^	Nanostructured Sr-doped HA	Chemical precipitation; nanopowder	Partial Sr substitution, approximately 15%	*S. aureus*, *Listeria monocytogenes* (*L. monocytogenes*), *Salmonella Enteritidis* (*S. Enteritidis*), *Acinetobacter baumannii* (*A. baumanii*)	At 100 mg/mL, Sr-HA significantly inhibited all tested strains except *E. coli*. The strongest antibacterial effect was observed against *A. baumannii*, with a 94.14% reduction in viable cells after 24 h.	[127]
Ce^3+^	Ce-substituted HA nanoparticles	Coprecipitation; nanoparticles	Ca_10−x_Ce_x_(PO_4_)_6_(OH)_2_, x = 0.01, 0.03, and 0.05	*S. aureus*, *E. coli*	The x = 0.03 and 0.05 compositions showed significantly greater antibacterial activity than pure HA and the x = 0.01 composition. Bacterial survival decreased with increasing Ce content, with a significant effect against *E. coli* at x = 0.05.	[141]
Ce^3+^	Ce-doped HA suspension and coating	Coprecipitated Ce-HA powder used as a suspension and as a sputtering target; RF magnetron sputtering on Ti	Ca_10−x_Ce_x_(PO_4_)_6_(OH)_2_, x = 0.05 (5% Ce substitution)	*S. aureus*, *E. coli*, *C. albicans*	Both the suspension and coating inhibited colony formation for all tested microorganisms. The suspension was more effective than the coating, and inhibition increased with exposure time; after 72 h, the suspension exhibited biocidal activity against *E. coli* and *C. albicans*.	[142]
Ce^3+^	Ce-HA-containing orthodontic adhesive	Ce-HA nanoparticles incorporated into dental adhesive	Ce in nanoparticles: 4.3 wt.% by EDAX; adhesive test concentrations: 20 and 40 µg/mL	*S. aureus*, *L. acidophilus*, *S. mutans*	The 40 µg/mL formulation showed the greatest activity, producing inhibition zones of 30 mm against *S. aureus*, 30 mm against L. acidophilus, and 25 mm against *S. mutans*.	[35]
Ce^3+^	Ce-substituted HA powder	Precipitation followed by calcination at 700 °C; nanopowder	Ce: 3, 5, and 7 mol% as Ce/(Ca + Ce)	*S. aureus*, *E. coli*, *P. aeruginosa*, *C. albicans*	The 5 mol% Ce-HA sample showed the strongest activity, with inhibition zones of 26 mm against *S. aureus*, 21 mm against *E. coli*, 25 mm against *P. aeruginosa*, and 25 mm against *C. albicans*.	[59]

**Table 2:** Representative commercial or clinically translated metal-containing HA products and technologies.

Product	Composition	Biomedical Application	Ref.
Acusure Ag^®^	Ag ions incorporated into an HA plasma-spray coating	Antimicrobial and osteoconductive coating for orthopedic implants	[182]
AG-PROTEX^®^	Ag-containing HA thermal-spray coating	Antibacterial coating for cementless joint prostheses	[183]
Resitage™	Spinal interbody cage coated with Ag-containing HA	Prevention of postoperative infection and support of spinal fusion	[184]

## Data Availability

No new data were created or analyzed in this study. Data sharing is not applicable to this article.

## References

[1] Eliaz N., Metoki N. (2017). Calcium phosphate bioceramics: A review of their history, structure, properties, coating technologies and biomedical applications. Materials.

[2] Jeong J., Kim J.H., Shim J.H., Hwang N.S., Heo C.Y. (2019). Bioactive calcium phosphate materials and applications in bone regeneration. Biomater. Res..

[3] Miroshnichenko L.A., Polyakova T.Y., Litvinova L.S., Khlusov I.A. (2024). Review of local cellular and molecular processes of bone tissue regeneration induced by calcium phosphate materials. Cell Tissue Biol..

[4] Liao H., Hoekstra J., Wolke J., Leeuwenburgh S.C.G., Jansen J. (2020). Injectable Calcium Phosphate Cements for the Reconstruction/Repair of Oral and Cranio-Maxillofacial Bone Defects: Clinical Outcome and Perspectives. Organ Tissue Engineering.

[5] Dewi A.H., Ana I.D. (2018). The Use of Hydroxyapatite Bone Substitute Grafting for Alveolar Ridge Preservation, Sinus Augmentation, and Periodontal Bone Defect: A Systematic Review. Heliyon.

[6] Izumi Y., Aoki A., Yamada Y., Kobayashi H., Iwata T., Akizuki T., Suda T., Nakamura S., Wara-Aswapati N., Ueda M. (2011). Current and Future Periodontal Tissue Engineering. Periodontology 2000.

[7] Sheikh Z., Hamdan N., Ikeda Y., Grynpas M.D., Ganss B., Glogauer M. (2017). Natural Graft Tissues and Synthetic Biomaterials for Periodontal and Alveolar Bone Reconstructive Applications: A Review. Biomater. Res..

[8] Cheah C.W., Al-Namnam N.M., Lau M.N., Lim G.S., Raman R.P.C., Fairbairn P., Ngeow W.C. (2021). Synthetic Material for Bone, Periodontal, and Dental Tissue Regeneration: Where Are We Now, and Where Are We Heading Next?. Materials.

[9] Alsahafi R., Mitwalli H., Balhaddad A.A., Weir M.D., Xu H.H.K., Melo M.A.S. (2021). Regenerating Craniofacial Dental Defects With Calcium Phosphate Cement Scaffolds: Current Status and Innovative Scope Review. Front. Dent. Med..

[10] Albee F.H. (1920). Studies in bone growth: Triple calcium phosphate as a stimulus to osteogenesis. Ann. Surg..

[11] Gaihre B., Uswatta S., Jayasuriya A.C. (2017). Reconstruction of Craniomaxillofacial Bone Defects Using Tissue-Engineering Strategies With Injectable and Non-Injectable Scaffolds. J. Funct. Biomater..

[12] Mondal S., Pal U. (2017). Recent Progress on Fabrication and Drug Delivery Applications of Nanostructured Hydroxyapatite. Wiley Interdiscip. Rev. Nanomed. Nanobiotechnol..

[13] Kumar P., Saini M., Dehiya B.S., Sindhu A., Kumar V., Kumar R., Lamberti L., Pruncu C.I., Thakur R. (2020). Comprehensive Survey on Nanobiomaterials for Bone Tissue Engineering Applications. Nanomaterials.

[14] Zhao R., Meng X., Pan Z., Li Y., Qian H., Zhu X., Yang X., Zhang X. (2025). Advancements in nanohydroxyapatite: Synthesis, biomedical applications and composite developments. Regen. Biomater..

[15] Cacciotti I. (2019). Multisubstituted Hydroxyapatite Powders and Coatings: The Influence of the Codoping on the Hydroxyapatite Performances. Int. J. Appl. Ceram. Technol..

[16] Radulescu D.-E., Vasile O.R., Andronescu E., Ficai A. (2023). Latest Research of Doped Hydroxyapatite for Bone Tissue Engineering. Int. J. Mol. Sci..

[17] Friederichs R.J., Chappell H.F., Shepherd D.V., Best S.M. (2015). Synthesis, characterization and modelling of zinc and silicate co-substituted hydroxyapatite. J. R. Soc. Interface.

[18] Zeglinski J., Nolan M., Bredol M., Schatte A., Tofail S.A.M. (2012). Unravelling the specific site preference in doping of calcium hydroxyapatite with strontium from ab initio investigations and Rietveld analyses. Phys. Chem. Chem. Phys..

[19] Santos R.D., Rezende M.V.D.S. (2014). Atomistic simulation of intrinsic defects and trivalent and tetravalent ion doping in hydroxyapatite. Adv. Condens. Matter Phys..

[20] Ullah I., Siddiqui M.A., Kolawole S.K., Liu H., Zhang J., Ren L., Yang K. (2020). Synthesis, characterization and in vitro evaluation of zinc and strontium binary doped hydroxyapatite for biomedical application. Ceram. Int..

[21] Saito T., Ishikawa Y., Noda Y., Yokoi T., Oshima Y., Nakamura A., Matsunaga K. (2023). Ca-vacancy effect on the stability of substitutional divalent cations in calcium-deficient hydroxyapatite. J. Am. Ceram. Soc..

[22] Guerra-López J.R., Bianchi A.E., Ramos M.A., Ferraresi-Curotto V., Güida J.A., Echeverría G.A. (2024). Novel synthesis and crystallographic results of zinc substituted hydroxyapatite with high thermal stability. Phys. B Condens. Matter.

[23] Lowry N., Han Y., Meenan B.J., Boyd A.R. (2017). Strontium and zinc co-substituted nanophase hydroxyapatite. Ceram. Int..

[24] Korneev A.V., Ryabchuk V.K., Kuz’mina M.A., Murzin P.D., Pavlychev A.A., Chelibanov V.P., Sturm E.V., Frank-Kamenetskaya O.V. (2024). Ti-Modified Hydroxyapatites: Synthesis, Crystal Chemistry, and Photocatalytic Activity. Cryst. Growth Des..

[25] Dhamale S.K., Honnungar S.S., Navalgund L., Jatti V.S. (2026). Correlating Phase Evolution and Morphology of Hydroxyapatite–Titanium Composites With Titanium Content and Thermal Processing. J. Eng. Mater. Technol..

[26] Wüster J., Neckel N., Sterzik F., Xiang-Tischhauser L., Barnewitz D., Genzel A., Koerdt S., Rendenbach C., Müller-Mai C., Heiland M. (2024). Effect of a synthetic hydroxyapatite-based bone grafting material compared to established bone substitute materials on regeneration of critical-size bone defects in the ovine scapula. Regen. Biomater..

[27] Liu W., Cheong N., He Z., Zhang T. (2025). Application of hydroxyapatite composites in bone tissue engineering: A review. J. Funct. Biomater..

[28] Kadirvelu L., Sivaramalingam S.S., Jothivel D., Chithiraiselvan D.D., Govindarajan D.K., Kandaswamy K. (2024). A review on antimicrobial strategies in mitigating biofilm-associated infections on medical implants. Curr. Res. Microb. Sci..

[29] Elsheikh R., Makram A., Toth L., Hirschmann M., Adam M. (2026). Comparative effectiveness of antimicrobial implant surface coatings in preventing orthopaedic implant-associated infections: A network meta-analysis. Arch. Orthop. Trauma Surg..

[30] Liu X., Ma P., Liu X., Liu S., Liu X., Wang R., Gao H. (2026). Resistance mechanisms of bacterial biofilms on orthopedic implants and research progress on novel anti-biofilm coatings. Front. Bioeng. Biotechnol..

[31] Imran E., Mei M.L., Li K.C., Ratnayake J., Ekambaram M., Cooper P.R. (2024). Dental applications of ion-substituted hydroxyapatite: A review of the literature. Dent. J..

[32] Wang X., Huang S., Peng Q. (2023). Metal ion-doped hydroxyapatite-based materials for bone defect restoration. Bioengineering.

[33] Pinchuk N.D., Piecuch A., Charczuk N., Sobierajska P., Targonska S., Bezkrovnyi O., Ogórek R., Wang Y., Wiglusz R.J. (2024). Effect of silver ion and silicate group on the antibacterial and antifungal properties of nanosized hydroxyapatite. Sci. Rep..

[34] Jamarun N., Afriska L.N., Wellia D.V., Prasejati A., Amirullah T.Y., Trycahyani N.A. (2024). Investigation of the antibacterial activity of synthesized hydroxyapatite Sr-doped nanocomposite. J. Appl. Pharm. Sci..

[35] Singh S., Jain R.K. (2024). The synthesis, characterization, and assessment of antibacterial properties of an orthodontic adhesive containing cerium-substituted hydroxyapatite nanoparticles: An in vitro study. Cureus.

[36] Weiss K.M., Kucko S.K., Mokhtari S., Keenan T.J., Wren A.W. (2023). Investigating the structure, solubility, and antibacterial properties of silver-and copper-doped hydroxyapatite. J. Biomed. Mater. Res. B Appl. Biomater..

[37] Wang R., Wang Y., Chen J., Yin Y., Zheng N., Li M., Zhao S., Han X., Li Q., Niu Y. (2025). Photocatalytic TiO 2/HAP nanocomposite for antimicrobial treatment, promineralization, and tooth whitening. RSC Adv..

[38] Lebedev V.N., Kharovskaya M.I., Lazoryak B.I., Solovieva A.O., Fadeeva I.V., Amirov A.A., Koliushenkov M.A., Orudzhev F.F., Baryshnikova O.V., Yankova V.G. (2024). Strontium and copper Co-doped multifunctional calcium phosphates: Biomimetic and antibacterial materials for bone implants. Biomimetics.

[39] Ishak A., Mazonakis N., Spernovasilis N., Akinosoglou K., Tsioutis C. (2025). Bactericidal versus bacteriostatic antibacterials: Clinical significance, differences and synergistic potential in clinical practice. J. Antimicrob. Chemother..

[40] Bellantone M., Williams H.D., Hench L.L. (2002). Broad-spectrum bactericidal activity of Ag_2_O-doped bioactive glass. Antimicrob. Agents Chemother..

[41] Arcos D., Vallet-Regí M. (2020). Substituted hydroxyapatite coatings of bone implants. J. Mater. Chem. B.

[42] Wang K., Zhou C., Hong Y., Zhang X. (2012). A review of protein adsorption on bioceramics. Interface Focus.

[43] Souza J.G.S., Bertolini M., Liu J., Nagay B.E., Martins R., Costa R.C., Brunson J.C., Shibli J., Figueiredo L.C., Dongari-Bagtzoglou A. (2025). Exploring the Impact of Biotic and Abiotic Surfaces on Protein Binding Modulation and Bacteria Attachment: Integrating Biological and Mathematical Approaches. ACS Nano.

[44] Zhang Y., Dai Z., Li X., He A., Zheng J., Ding M., Li Q., Mou Y., Yang D., Xiu W. (2026). Emerging non-antibiotic strategies for implant-associated biofilm infections by reprogramming the dysregulated immune microenvironment. npj Biofilms Microbiomes.

[45] Bohara S., Suthakorn J. (2022). Surface coating of orthopedic implant to enhance the osseointegration and reduction of bacterial colonization: A review. Biomater. Res..

[46] Xiang B., Jiang J., Wang H., Song L. (2025). Research progress of implantable materials in antibacterial treatment of bone infection. Front. Bioeng. Biotechnol..

[47] Hoveidaei A.H., Sabaghian A., Basirat E., Ramezani A., Shu H.T., Conway J.D. (2025). Local Antibiotic Delivery Systems and Their Applications in Orthopaedic Surgery. JBJS Open Access.

[48] Lubojański A., Zakrzewski W., Samól K., Bieszczad-Czaja M., Świtała M., Wiglusz R., Watras A., Mielan B., Dobrzyński M. (2024). Application of nanohydroxyapatite in medicine—A narrative review. Molecules.

[49] Popowski W., Koseski D., Domanowska D., Zalewska M., Popowska M. (2025). Bacterial colonization of bone substitute materials used in oral surgery: Mechanisms, clinical implications, and preventive strategies—A narrative review. Front. Microbiol..

[50] Yilmaz B., Alshemary A.Z., Evis Z. (2019). Co-doped hydroxyapatites as potential materials for biomedical applications. Microchem. J..

[51] Mocanu A., Cadar O., Frangopol P.T., Petean I., Tomoaia G., Paltinean G.-A., Racz C.P., Horovitz O., Tomoaia-Cotisel M. (2021). Ion release from hydroxyapatite and substituted hydroxyapatites in different immersion liquids: In vitro experiments and theoretical modelling study. R. Soc. Open Sci..

[52] Mondal S., Park S., Choi J., Vu T.T.H., Doan V.H.M., Vo T.T., Lee B., Oh J. (2023). Hydroxyapatite: A journey from biomaterials to advanced functional materials. Adv. Colloid Interface Sci..

[53] Liu Y., Nadeem A., Sebastian S., Olsson M.A., Wai S.N., Styring E., Engellau J., Isaksson H., Tägil M., Lidgren L. (2022). Bone mineral: A trojan horse for bone cancers. Efficient mitochondria targeted delivery and tumor eradication with nano hydroxyapatite containing doxorubicin. Mater. Today Bio.

[54] Lara-Ochoa S., Ortega-Lara W., Guerrero-Beltrán C.E. (2021). Hydroxyapatite nanoparticles in drug delivery: Physicochemistry and applications. Pharmaceutics.

[55] Safitri N., Rauf N., Tahir D. (2023). Enhancing drug loading and release with hydroxyapatite nanoparticles for efficient drug delivery: A review synthesis methods, surface ion effects, and clinical prospects. J. Drug Deliv. Sci. Technol..

[56] Singh L., Jain H., Sharma P., Singh M., Ezebuiro V. (2026). Functionalized hydroxyapatite nanocomposites for localized drug delivery in bone cancer. Hybrid Adv..

[57] Devanand Venkatasubbu G., Ramasamy S., Ramakrishnan V., Kumar J. (2011). Nanocrystalline hydroxyapatite and zinc-doped hydroxyapatite as carrier material for controlled delivery of ciprofloxacin. 3 Biotech.

[58] Iconaru S.L., Predoi D., Ciobanu C.S., Negrila C.C., Trusca R., Raaen S., Rokosz K., Ghegoiu L., Badea M.L., Cimpeanu C. (2024). Novel Antimicrobial Agents Based on Zinc-Doped Hydroxyapatite Loaded with Tetracycline. Antibiotics.

[59] Moaness M., Mousa S.M., Abo-Elfadl M.T., El-Bassyouni G.T. (2024). Doxorubicin loaded cerium substituted hydroxyapatite nanoparticles: A promising new therapeutic approach for bone regeneration, doxorubicin delivery, and cancer treatment. Int. J. Pharm..

[60] Maggi L., Friuli V., Cerea B., Bruni G., Berbenni V., Bini M. (2024). Physicochemical characterization of hydroxyapatite hybrids with meloxicam for dissolution rate improvement. Molecules.

[61] Sprio S., Preti L., Montesi M., Panseri S., Adamiano A., Vandini A., Pugno N.M., Tampieri A. (2019). Surface Phenomena Enhancing the Antibacterial and Osteogenic Ability of Nanocrystalline Hydroxyapatite, Activated by Multiple-Ion Doping. ACS Biomater. Sci. Eng..

[62] Zhu H., Guo D., Sun L., Li H., Hanaor D.A.H., Schmidt F., Xu K. (2018). Nanostructural insights into the dissolution behavior of Sr-doped hydroxyapatite. J. Eur. Ceram. Soc..

[63] Yin I.X., Zhang J., Zhao I.S., Mei M.L., Li Q., Chu C.H. (2020). The antibacterial mechanism of silver nanoparticles and its application in dentistry. Int. J. Nanomed..

[64] Ma X., Zhou S., Xu X., Du Q. (2022). Copper-containing nanoparticles: Mechanism of antimicrobial effect and application in dentistry-a narrative review. Front. Surg..

[65] More P.R., Pandit S., Filippis A.D., Franci G., Mijakovic I., Galdiero M. (2023). Silver nanoparticles: Bactericidal and mechanistic approach against drug resistant pathogens. Microorganisms.

[66] Mosina M., Siverino C., Stipniece L., Sceglovs A., Vasiljevs R., Moriarty T.F., Locs J. (2023). Gallium-doped hydroxyapatite shows antibacterial activity against pseudomonas aeruginosa without affecting cell metabolic activity. J. Funct. Biomater..

[67] Li F., Liu F., Huang K., Yang S. (2022). Advancement of gallium and gallium-based compounds as antimicrobial agents. Front. Bioeng. Biotechnol..

[68] Hassanain M., Abdel-Ghafar H.M., Hamouda H.I., El-Hosiny F.I., Ewais E.M. (2024). Enhanced antimicrobial efficacy of hydroxyapatite-based composites for healthcare applications. Sci. Rep..

[69] Sadovnikova M.A., Shurtakova D.V., Mamin G.V., Murzakhanov F.F., Zobkova Y.O., Petrakova N.V., Komlev V.S., Gafurov M.R. (2025). Ce (III) Ions in Hydroxyapatite: Nanoscale Environment Investigation. Appl. Magn. Reson..

[70] Rodrigues A.S., Batista J.G., Rodrigues M.Á., Thipe V.C., Minarini L.A., Lopes P.S., Lugão A.B. (2024). Advances in silver nanoparticles: A comprehensive review on their potential as antimicrobial agents and their mechanisms of action elucidated by proteomics. Front. Microbiol..

[71] Kucukosman O.K., Pourmostafa A., Dogan E., Bensalem A. (2025). Balancing antibacterial activity and toxicity in silver-loaded hydroxyapatite: The impact of the silver nanoparticle incorporation method. J. Mater. Chem. B.

[72] Ciobanu C.S., Iconaru S.L., Le Coustumer P., Constantin L.V., Predoi D. (2012). Antibacterial activity of silver-doped hydroxyapatite nanoparticles against gram-positive and gram-negative bacteria. Nanoscale Res. Lett..

[73] Silhavy T., Kahne D., Walker S. (2010). The bacterial cell envelope. Cold Spring Harb. Perspect. Biol..

[74] Abbaszadegan A., Ghahramani Y., Gholami A., Hemmateenejad B., Dorostkar S., Nabavizadeh M., Sharghi H. (2015). The effect of charge at the surface of silver nanoparticles on antimicrobial activity against gram-positive and gram-negative bacteria: A preliminary study. J. Nanomater..

[75] Taglietti A., Díaz Fernández Y., Amato E., Cucca L., Dacarro G., Grisoli P., Necchi V., Pallavicini P., Pasotti L., Patrini M. (2012). Antibacterial activity of glutathione-coated silver nanoparticles against Gram positive and Gram negative bacteria. Langmuir ACS J. Surf. Colloids.

[76] Ouay B.L., Stellacci F. (2015). Antibacterial activity of silver nanoparticles: A surface science insight. Nano Today.

[77] Sinulingga K., Sirait M., Siregar N., Doloksaribu M.E. (2021). Investigation of antibacterial activity and cell viability of Ag/Mg and Ag/Zn co-doped hydroxyapatite derived from natural limestone. ACS Omega.

[78] Kontakis M.G., Carlsson E., Palo-Nieto C., Hailer N.P. (2025). Ionic silver coating of orthopedic implants may impair osteogenic differentiation and mineralization. Exp. Ther. Med..

[79] Gokcekaya O., Ergun C., Webster T., Bahadir A., Ueda K., Narushima T., Nakano T. (2021). Effect of Precursor Deficiency Induced Ca/P Ratio on Antibacterial and Osteoblast Adhesion Properties of Ag-Incorporated Hydroxyapatite: Reducing Ag Toxicity. Materials.

[80] Bai X., Sandukas S., Appleford M., Ong J., Rabiei A. (2012). Antibacterial effect and cytotoxicity of Ag-doped functionally graded hydroxyapatite coatings. J. Biomed. Mater. Res. B Appl. Biomater..

[81] Suchánková L., Kvítek L., Kolář M., Panáček A. (2026). Emerging strategies of bacterial adaptation mechanisms to silver and metal oxide nanomaterials. FEMS Microbiol. Rev..

[82] Jacobs A., Renaudin G., Forestier C., Nedelec J., Descamps S. (2020). Biological properties of copper-doped biomaterials for orthopedic applications: A review of antibacterial, angiogenic and osteogenic aspects. Acta Biomater..

[83] Noori A., Hoseinpour M., Kolivand S., Lotfibakhshaiesh N., Ebrahimi-Barough S., Ai J., Azami M. (2024). Exploring the various effects of Cu doping in hydroxyapatite nanoparticle. Sci. Rep..

[84] Ciobanu C.S., Predoi D., Iconaru S.L., Predoi M.V., Ghegoiu L., Buton N., Motelica-Heino M. (2024). Copper doped hydroxyapatite nanocomposite thin films: Synthesis, physico–chemical and biological evaluation. Biometals.

[85] Tuntun S.M., Hossain M.S., Uddin M.N., Shaikh M.A.A., Bahadur N.M., Ahmed S. (2023). Crystallographic characterization and application of copper doped hydroxyapatite as a biomaterial. New J. Chem..

[86] National Institutes of Health, Office of Dietary Supplements (2022). Copper: Fact Sheet for Health Professionals. https://ods.od.nih.gov/factsheets/Copper-HealthProfessional/.

[87] Eremina N.V., Bulina N.V., Mikhailenko M.A., Vinokurova O.B., Prosanov I.Y., Chaikina M.V. (2023). Cu-substituted hydroxyapatite powder: Mechanochemical synthesis using different copper sources and thermal stability. Powders.

[88] Ramos-Zúñiga J., Bruna N., Pérez-Donoso J. (2023). Toxicity Mechanisms of Copper Nanoparticles and Copper Surfaces on Bacterial Cells and Viruses. Int. J. Mol. Sci..

[89] Saraeva I., Tolordava E., Yushina Y., Sozaev I., Sokolova V., Khmelnitskiy R., Sheligyna S., Pallaeva T., Pokryshkin N., Khmelenin D. (2022). Direct Bactericidal Comparison of Metal Nanoparticles and Their Salts against S. aureus Culture by TEM and FT-IR Spectroscopy. Nanomaterials.

[90] Chang T., Babu R., Zhao W., Johnson C., Hedström P., Odnevall I., Leygraf C. (2021). High-Resolution Microscopical Studies of Contact Killing Mechanisms on Copper-Based Surfaces. ACS Appl. Mater. Interfaces.

[91] Wang W., Cui Y., Wei X., Zang Y., Chen X., Cheng L., Wang X. (2024). CuCo_2_O_4_ Nanoflowers with Multiple Enzyme Activities for Treating Bacterium-Infected Wounds via Cuproptosis-like Death. ACS Nano.

[92] Li M., Zhu Y., Xia H., Yao M., Chu X., Wang X., Yang K., Yang M., Zhang Y., Mao C. (2015). Towards a Molecular Understanding of the Antibacterial Mechanism of Copper-bearing Titanium Alloys against Staphylococcus aureus. Adv. Healthc. Mater..

[93] Liu Y., Nie N., Tang H., Zhang C., Chen K., Wang W., Liu J. (2021). Effective Antibacterial Activity of Degradable Copper-Doped Phosphate-Based Glass Nanozymes. ACS Appl. Mater. Interfaces.

[94] Pramanik A., Laha D., Bhattacharya D., Pramanik P., Karmakar P. (2012). A novel study of antibacterial activity of copper iodide nanoparticle mediated by DNA and membrane damage. Colloids Surf. B Biointerfaces.

[95] Zheng J., Wang Y., Lü J. (2025). Unexpected Enhancement of the Antibacterial Activity of Copper Ions in the Presence of Nanobubbles. Langmuir ACS J. Surf. Colloids.

[96] Stanić V., Dimitrijević S., Antić-Stanković J., Mitrić M., Jokić B., Plećaš I.B., Raičević S. (2010). Synthesis, characterization and antimicrobial activity of copper and zinc-doped hydroxyapatite nanopowders. Appl. Surf. Sci..

[97] Benali Y., Predoi D., Rokosz K., Ciobanu C.S., Iconaru S.L., Raaen S., Negrila C.C., Cimpeanu C., Trusca R., Ghegoiu L. (2024). Physico-chemical properties of copper-doped hydroxyapatite coatings obtained by vacuum deposition technique. Materials.

[98] Prosolov K., Lastovka V., Khimich M., Glukhov I., Kashin A., Luginin N., Sharkeev Y. (2023). Influence of Cu Substitution on the Properties of Hydroxyapatite Targets and Deposited Coatings. Coatings.

[99] Korowash S.I., Keskin-Erdogan Z., Hemdan B.A., Barrios Silva L.V., Ibrahim D.M., Chau D.Y. (2023). Selenium-and/or copper-substituted hydroxyapatite: A bioceramic substrate for biomedical applications. J. Biomater. Appl..

[100] National Institutes of Health, Office of Dietary Supplements Zinc: Fact Sheet for Health Professionals. https://ods.od.nih.gov/factsheets/Zinc-HealthProfessional/.

[101] Wen X., Wang J., Pei X., Zhang X. (2023). Zinc-based biomaterials for bone repair and regeneration: Mechanism and applications. J. Mater. Chem. B.

[102] Molenda M., Kolmas J. (2023). The role of zinc in bone tissue health and regeneration—A review. Biol. Trace Elem. Res..

[103] Dornelas J., Dornelas G., Rossi A., Piattelli A., Di Pietro N., Romasco T., Mourão C.F., Alves G.G. (2024). The incorporation of zinc into hydroxyapatite and its influence on the cellular response to biomaterials: A systematic review. J. Funct. Biomater..

[104] Fosca M., Streza A., Antoniac I.V., Vadalà G., Rau J.V. (2023). Ion-doped calcium phosphate-based coatings with antibacterial properties. J. Funct. Biomater..

[105] Guerra-López J.R., Bianchi A.E., Ramos M.A., Ubertino M., Ferraresi-Curotto V., Güida J.A., Barbaro K., Zhukova A.A., Grigorieva V.Y., Rau J.V. (2025). Preparation of Zinc-doped hydroxyapatite ceramics and evaluation of biocompatibility and antibacterial activity. J. Funct. Biomater..

[106] Predoi D., Iconaru S.L., Predoi M.V., Motelica-Heino M., Guegan R., Buton N. (2019). Evaluation of antibacterial activity of zinc-doped hydroxyapatite colloids and dispersion stability using ultrasounds. Nanomaterials.

[107] Sirelkhatim A., Mahmud S., Seeni A., Kaus N.H.M., Ann L.C., Bakhori S.K.M., Hasan H., Mohamad D. (2015). Review on zinc oxide nanoparticles: Antibacterial activity and toxicity mechanism. Nano-Micro Lett..

[108] Cuypers L.A., de Boer L., Wang R., Walboomers X.F., Yang F., Zaat S.A., Leeuwenburgh S.C. (2024). Antibacterial activity of zinc-doped hydroxyapatite and vancomycin-loaded gelatin nanoparticles against intracellular staphylococcus aureus in human thp-1 derived macrophages. ACS Appl. Nano Mater..

[109] Fatimah I., Hidayat H., Citradewi P.W., Tamyiz M., Doong R., Sagadevan S. (2023). Hydrothermally synthesized titanium/hydroxyapatite as photoactive and antibacterial biomaterial. Heliyon.

[110] Li Y., Ho J., Ooi C.P. (2010). Antibacterial efficacy and cytotoxicity studies of copper (II) and titanium (IV) substituted hydroxyapatite nanoparticles. Mater. Sci. Eng. C.

[111] Liu L., Huang R., Zhang L. (2020). Cobalt element doping for biomedical use: A review. Mater. Sci. Forum.

[112] Office of Dietary Supplements National Institutes of Health Vitamin B12 Fact Sheet for Health Professionals. https://ods.od.nih.gov/factsheets/VitaminB12-HealthProfessional/.

[113] Tank K.P., Chudasama K.S., Thaker V.S., Joshi M.J. (2013). Cobalt-doped nanohydroxyapatite: Synthesis, characterization, antimicrobial and hemolytic studies. J. Nanopart. Res..

[114] Yazdani N., Javadpour J., Eftekhari Y.B., Hamrang M. (2019). Hydrothermal Synthesis of Cobalt-Doped Hydroxyapatite Nanoparticles: Structure, Magnetic Behaviour, Bioactivity and Antibacterial Activity. Iran. J. Mater. Sci. Eng..

[115] Kulanthaivel S., Roy B., Agarwal T., Giri S., Pramanik K., Pal K., Ray S.S., Maiti T.K., Banerjee I. (2016). Cobalt doped proangiogenic hydroxyapatite for bone tissue engineering application. Mater. Sci. Eng. C.

[116] Yan L., Wei X., Zhang Z., Wang C., Jia Y., Wang L., Yan Y., Fan X. (2024). Cobalt-doped hydroxyapatite for bone tissue engineering: Synthesis, characterization and in vitro biocompatibility of real-time extract. Mater. Today Commun..

[117] Safari-Gezaz M., Parhizkar M., Asghari E. (2025). Effect of cobalt ions doping on morphology and electrochemical properties of hydroxyapatite coatings for biomedical applications. Sci. Rep..

[118] Yan N., Zhou H., Jin P., Li T., Liu Q., Ning H., Ma Z., Feng L., Jin T., Deng Y. (2025). A Multifunctional Cobalt-Containing Implant for Treating Biofilm Infections and Promoting Osteointegration in Infected Bone Defects Through Macrophage-Mediated Immunomodulation. Adv. Sci..

[119] Choi S., Hassan M.A., Britigan B.E., Narayanasamy P. (2024). Antimicrobial activity of gallium (III) compounds: Pathogen-dependent targeting of multiple iron/heme-dependent biological processes. Curr. Issues Mol. Biol..

[120] Ikhazuagbe I.H., Ofoka E.A., Odofin O.L., Erumiseli O., Edoka O.E., Ezennubia K.P., Ogbunike P.M., Annan E., Okonkwo S.S., Dike J.C. (2025). Antibacterial activity and mechanistic insights of gallium-based nanoparticles: An emerging frontier in metal-based antimicrobials. RSC Adv..

[121] Pajor K., Michalicha A., Belcarz A., Pajchel L., Zgadzaj A., Wojas F., Kolmas J. (2022). Antibacterial and cytotoxicity evaluation of new hydroxyapatite-based granules containing silver or gallium ions with potential use as bone substitutes. Int. J. Mol. Sci..

[122] Kurtjak M., Vukomanović M., Krajnc A., Kramer L., Turk B., Suvorov D. (2016). Designing Ga (iii)-containing hydroxyapatite with antibacterial activity. RSC Adv..

[123] Almuradova E., Cicin I. (2023). Cancer-related hypercalcemia and potential treatments. Front. Endocrinol..

[124] You J., Zhang Y., Zhou Y. (2022). Strontium functionalized in biomaterials for bone tissue engineering: A prominent role in osteoimmunomodulation. Front. Bioeng. Biotechnol..

[125] Akobundu U.U., Ifijen I.H., Duru P., Igboanugo J.C., Ekanem I., Fagbolade M., Ajayi A.S., George M., Atoe B., Matthews J.T. (2025). Exploring the role of strontium-based nanoparticles in modulating bone regeneration and antimicrobial resistance: A public health perspective. RSC Adv..

[126] Qiao J., Wu G., Jiang S., Yong Z., Ma F., Jiao J. (2025). Synthesis, surface chemical characterization, and enhanced osteoblast response of strontium-substituted hydroxyapatite nanoparticles for alveolar bone regeneration. J. Nanostruct. Chem..

[127] Mirković M., Sknepnek A., Kalijadis A., Krstić A., Šuljagić M., Perić M., Andjelković L. (2025). Nanostructured Sr-Doped Hydroxyapatite: A Material with Antimicrobial Potential. Nanomaterials.

[128] Li Y., Wang W., Han J., Li Z., Wang Q., Lin X., Ge K., Zhou G. (2022). Synthesis of Silver-and Strontium-Substituted Hydroxyapatite with Combined Osteogenic and Antibacterial Activities. Biol. Trace Elem. Res..

[129] Capuccini C., Torricelli P., Sima F., Boanini E., Ristoscu C., Bracci B., Socol G., Fini M., Mihăilescu I., Bigi A. (2008). Strontium-substituted hydroxyapatite coatings synthesized by pulsed-laser deposition: In vitro osteoblast and osteoclast response. Acta Biomater..

[130] Lourenço A., Torres A., Vasconcelos D., Ribeiro-Machado C., Barbosa J., Barbosa M., Barrias C., Ribeiro C. (2019). Osteogenic, anti-osteoclastogenic and immunomodulatory properties of a strontium-releasing hybrid scaffold for bone repair. Mater. Sci. Eng. C Mater. Biol. Appl..

[131] Lu T., Zhang L., Yuan X., Ye J. (2024). A novel calcium phosphate-based ceramic scaffolds with unexpected high osteogenic activity by strontium doping. Mater. Today Chem..

[132] Stipniece L., Wilson S., Curran J., Chen R., Salma-Ancane K., Sharma P., Meenan B., Boyd A. (2020). Strontium substituted hydroxyapatite promotes direct primary human osteoblast maturation. Ceram. Int..

[133] Sullivan C.O., Neill O., O’ Leary N., Gara J.O., Crean A., Ryan K. (2021). Osteointegration, antimicrobial and antibiofilm activity of orthopaedic titanium surfaces coated with silver and strontium-doped hydroxyapatite using a novel blasting process. Drug Deliv. Transl. Res..

[134] Maqbool M., Nawaz Q., Rehman M.A.U., Cresswell M., Jackson P., Hurle K., Detsch R., Goldmann W., Shah A., Boccaccini A. (2021). Synthesis, Characterization, Antibacterial Properties, and In Vitro Studies of Selenium and Strontium Co-Substituted Hydroxyapatite. Int. J. Mol. Sci..

[135] Lu T., Li F., He W., Wang Y., Shi W., Jiang J., Chen X. (2025). Preparation and characterization of superhydrophilic Sr-doped hydroxyapatite coatings for medical implant surfaces. Ceram. Int..

[136] Liu L., Hou S., Xu G., Gao J., Mu J., Gao M., He J., Su X., Yang Z., Liu Y. (2024). Evaluation of osteogenic properties of a novel injectable bone-repair material containing strontium in vitro and in vivo. Front. Bioeng. Biotechnol..

[137] Huang Y., Zhang Y., Li M., Yang H., Liang J., Chen Y., Zhang Y., Huang X., Xie L., Lin H. (2022). Physicochemical, osteogenic and antimicrobial properties of graphene oxide reinforced silver/strontium-doped hydroxyapatite on titanium for potential orthopedic applications. Surf. Coat. Technol..

[138] Fielding G., Roy M., Bandyopadhyay A., Bose S. (2012). Antibacterial and biological characteristics of plasma sprayed silver and strontium doped hydroxyapatite coatings. Acta Biomater..

[139] Chen S., Wang Y., Bao S., Yao L., Fu X., Yu Y., Lyu H., Pang H., Guo S., Zhang H. (2024). Cerium oxide nanoparticles in wound care: A review of mechanisms and therapeutic applications. Front. Bioeng. Biotechnol..

[140] Bai Y., Li Y., Li Y., Tian L. (2024). Advanced biological applications of cerium oxide nanozymes in disease related to oxidative damage. ACS Omega.

[141] Ciobanu C., Popa C., Predoi D. (2016). Cerium doped hydroxyapatite nanoparticles synthesized by coprecipitation method. J. Serbian Chem. Soc..

[142] Predoi D., Iconaru S.L., Predoi M.V., Groza A., Gaiaschi S., Rokosz K., Raaen S., Negrila C.C., Prodan A.-M., Costescu A. (2020). Development of cerium-doped hydroxyapatite coatings with antimicrobial properties for biomedical applications. Coatings.

[143] Ivankovic T., Zuzic A., Urlic I., Gobbo V.A., Dadic B., Sandblom T., Zakeri S., Virpinen M., Goic-Barisic I., Hrenovic J. (2025). Cerium, copper, gallium, manganese and silver substituted hydroxyapatite: Cytotoxicity and antibacterial activity toward multidrug-resistant clinical isolates. J. Hazard. Mater. Adv..

[144] Kubiak-Mihkelsoo Z., Kostrzębska A., Błaszczyszyn A., Pitułaj A., Dominiak M., Gedrange T., Nawrot-Hadzik I., Matys J., Hadzik J. (2025). Ionic doping of hydroxyapatite for bone regeneration: Advances in structure and properties over two decades—A narrative review. Appl. Sci..

[145] Kolmas J., Groszyk E., Kwiatkowska-Różycka D. (2014). Substituted hydroxyapatites with antibacterial properties. BioMed Res. Int..

[146] Jadbabaee S., Mohebi Far F., Esmaeili J., Kolahdoozan M. (2025). The role of ion-doped hydroxyapatite in drug delivery, tissue engineering, wound healing, implants, and imaging. Chemistry.

[147] Sevencan A., Doyuk E.K., Köse N. (2020). Silver ion doped hydroxyapatite-coated titanium pins prevent bacterial colonization. Jt. Dis. Relat. Surg..

[148] Jelínek M., Kocourek T., Remsa J., Weiserová M., Jurek K., Miksovsky J., Strnad J., Galandáková A., Ulrichová J. (2013). Antibacterial, cytotoxicity and physical properties of laser--silver doped hydroxyapatite layers. Mater. Sci. Eng. C Mater. Biol. Appl..

[149] Sivaraj D., Vijayalakshmi K. (2019). Enhanced antibacterial and corrosion resistance properties of Ag substituted hydroxyapatite/functionalized multiwall carbon nanotube nanocomposite coating on 316L stainless steel for biomedical application. Ultrason. Sonochem..

[150] Geng Z., Wang R., Zhuo X., Li Z., Huang Y., Ma L., Cui Z., Zhu S., Liang Y., Liu Y. (2017). Incorporation of silver and strontium in hydroxyapatite coating on titanium surface for enhanced antibacterial and biological properties. Mater. Sci. Eng. C Mater. Biol. Appl..

[151] Alshemary A.Z., Hussain R., Dalgic A.D., Evis Z. (2022). Bactericidal and in vitro osteogenic activity of nano sized cobalt-doped silicate hydroxyapatite. Ceram. Int..

[152] Gupta A., Verma M., Anand M., Sengupta P., Saravanan M., Manna I., Balani K. (2019). Antibacterial and magnetic response of site-specific cobalt incorporated hydroxyapatite. Ceram. Int..

[153] Ballardini A., Montesi M., Panseri S., Vandini A., Balboni P.G., Tampieri A., Sprio S. (2018). New hydroxyapatite nanophases with enhanced osteogenic and anti-bacterial activity. J. Biomed. Mater. Res. A.

[154] Zhong Z., Wu X., Wang Y., Li M., Li Y., Liu X., Zhang X., Lan Z., Wang J., Du Y. (2021). Zn/Sr dual ions-collagen co-assembly hydroxyapatite enhances bone regeneration through procedural osteo-immunomodulation and osteogenesis. Bioact. Mater..

[155] Zhu Z., Khodaei A., King H., Weinans H., Yavari A. (2026). Harnessing copper–strontium synergy in an injectable composite for multi-pathway bone regeneration. RSC Adv..

[156] Zhang H., Wu Z., Wang Z., Yan X., Duan X., Sun H. (2025). Advanced surface modification techniques for titanium implants: A review of osteogenic and antibacterial strategies. Front. Bioeng. Biotechnol..

[157] Dubey A., Ghosh S., Jaiswal S., Roy P., Lahiri D. (2022). Assessment of protein adhesion behaviour and biocompatibility of magnesium/Co-substituted HA-based composites for orthopaedic application. Int. J. Biol. Macromol..

[158] Liu Z., Yamada S., Otsuka Y., Galindo T.G.P., Tagaya M. (2022). Surface modification of hydroxyapatite nanoparticles for bone regeneration by controlling their surface hydration and protein adsorption states. Dalton Trans..

[159] Dorm B., Iemma M., Neto B.D., Francisco R.C.L., Dinić I., Ignjatović N., Marković S., Vukovic M., Škapin S., Trovatti E. (2022). Synthesis and Biological Properties of Alanine-Grafted Hydroxyapatite Nanoparticles. Life.

[160] Zhou J., Zhang F., Tang Q., Zhu T., Ni Y., Wu Q., Liu Q., Zhu R., Wang T., Zhang Y. (2024). Deoxygenated hydroxyapatite inhibits macrophage inflammation through fibronectin restricted adsorption. Acta Biomater..

[161] Chen M., Sun Y., Hou Y., Luo Z., Li M., Wei Y., Chen M., Tan L., Cai K., Hu Y. (2022). Constructions of ROS-responsive titanium-hydroxyapatite implant for mesenchymal stem cell recruitment in peri-implant space and bone formation in osteoporosis microenvironment. Bioact. Mater..

[162] Dias A.M., Canhas I.D.N., Bruziquesi C.G.O., Speziali M., Sinisterra R., Cortés M. (2022). Magnesium (Mg2 +), Strontium (Sr2 +), and Zinc (Zn2 +) Co-substituted Bone Cements Based on Nano-hydroxyapatite/Monetite for Bone Regeneration. Biol. Trace Elem. Res..

[163] Bernardo M., Da Silva B., Hamouda A., De Toledo M., Schalla C., Rütten S., Goetzke R., Mattoso L., Zenke M., Sechi A. (2022). PLA/Hydroxyapatite scaffolds exhibit in vitro immunological inertness and promote robust osteogenic differentiation of human mesenchymal stem cells without osteogenic stimuli. Sci. Rep..

[164] Tan L., Hu Y., Li M., Zhang Y., Xue C., Chen M., Luo Z., Cai K. (2021). Remotely-activatable extracellular matrix-mimetic hydrogel promotes physiological bone mineralization for enhanced cranial defect healing. Chem. Eng. J..

[165] Jin P., Liu L., Chen X., Xi S., Jiang T. (2023). Calcium-to-phosphorus releasing ratio affects osteoinductivity and osteoconductivity of calcium phosphate bioceramics in bone tissue engineering. Biomed. Eng. OnLine.

[166] Panzavolta S., Torricelli P., Casolari S., Parrilli A., Fini M., Bigi A. (2018). Strontium-Substituted Hydroxyapatite-Gelatin Biomimetic Scaffolds Modulate Bone Cell Response. Macromol. Biosci..

[167] Cirillo M., Martelli G., Boanini E., Rubini K., Di Filippo M., Torricelli P., Pagani S., Fini M., Bigi A., Giacomini D. (2021). Strontium substituted hydroxyapatite with β-lactam integrin agonists to enhance mesenchymal cells adhesion and to promote bone regeneration. Colloids Surf. B Biointerfaces.

[168] Li J., Zhang C., Li J., Gao R., Yang M., Yu L., Zhang W., Zhou G., Shen W., Zhang J. (2025). Strontium-incorporated hydroxyapatite nanocomposites promoting bone formation and angiogenesis by modulating M2 macrophage polarization in the bone microenvironment. Regen. Biomater..

[169] Durairaj S., Arora M., Kumar S., Murugesan P., Vivanco J., Ghosh D. (2026). Engineering osteoinductive hydroxyapatite via zinc and strontium co-substitution. Nanoscale.

[170] Silingardi F., Salamanna F., Espanol M., Maglio M., Sartori M., Giavaresi G., Bigi A., Ginebra M., Boanini E. (2024). Regulation of osteogenesis and angiogenesis by cobalt, manganese and strontium doped apatitic materials for functional bone tissue regeneration. Biomater. Adv..

[171] Biomaterialia A., Jiménez-Holguín J., Lozano D., Saiz-Pardo M., Pablo D., Ortega L., Enciso S., Fernández-Tomé B., Díaz-Güemes I., Sánchez-Margallo F. (2024). Osteogenic-angiogenic coupled response of cobalt-containing mesoporous bioactive glasses in vivo. Acta Biomater..

[172] Bosch-Rué È., Díez-Tercero L., Rodríguez-González R., Bosch-Canals B.M., Pérez R. (2021). Assessing the potential role of copper and cobalt in stimulating angiogenesis for tissue regeneration. PLoS ONE.

[173] Chai Y., Mendes L., Van Gastel N., Carmeliet G., Luyten F. (2018). Fine-tuning pro-angiogenic effects of cobalt for simultaneous enhancement of vascular endothelial growth factor secretion and implant neovascularization. Acta Biomater..

[174] Lin Z., Cao Y., Zou J.-M., Zhu F., Gao Y., Zheng X., Wang H., Zhang T., Wu T. (2020). Improved osteogenesis and angiogenesis of a novel copper ions doped calcium phosphate cement. Mater. Sci. Eng. C Mater. Biol. Appl..

[175] Sun Y., Liu X., Zhu Y., Han Y., Shen J., Bao B., Gao T., Lin J., Huang T., Xu J. (2021). Tunable and Controlled Release of Cobalt Ions from Metal-Organic Framework Hydrogel Nanocomposites Enhances Bone Regeneration. ACS Appl. Mater. Interfaces.

[176] Huang S.-M., Chen W.-C., Liu S.-M., Ko C.-L., Chen J.-C., Shih C. (2024). Insights into the Various Cellular Antimicrobial Responses, Biocompatibility, Osteogenesis, Wound Healing, and Angiogenesis of Copper-Doped Nano-Hydroxyapatite Composite Calcium Phosphate Bone Cement In Vitro. J. Compos. Sci..

[177] Bang L., Ramesh S., Purbolaksono J., Ching Y., Long B., Chandran H., Othman R. (2015). Effects of silicate and carbonate substitution on the properties of hydroxyapatite prepared by aqueous co-precipitation method. Mater. Des..

[178] Bigi A., Boanini E., Gazzano M. (2016). Ion substitution in biological and synthetic apatites. Biomineralization and Biomaterials, Fundamentals and Applications.

[179] Kurzyk A., Szwed-Georgiou A., Pagacz J., Antosik A., Tymowicz-Grzyb P., Gerle A., Szterner P., Włodarczyk M., Płociński P., Urbaniak M. (2023). Calcination and ion substitution improve physicochemical and biological properties of nanohydroxyapatite for bone tissue engineering applications. Sci. Rep..

[180] Tite T., Popa A., Balescu L., Bogdan I., Pasuk I., Ferreira J., Stan G. (2018). Cationic Substitutions in Hydroxyapatite: Current Status of the Derived Biofunctional Effects and Their In Vitro Interrogation Methods. Materials.

[181] Xia W., Lindahl C., Persson C., Thomsen P., Lausmaa J., Engqvist H. (2010). Changes of Surface Composition and Morphology after Incorporation of Ions into Biomimetic Apatite Coating. J. Biomater. Nanobiotechnol..

[182] Tabrizi E., Li B. (2025). Silver integrated hybrids and nanocomposites for next-generation biomedicine: Beyond antimicrobial coatings toward smart sense–response–heal platforms. Mater. Today Bio.

[183] Kawano S., Ueno M., Fujii M., Mawatari D., Mawatari M. (2022). Case Series of Silver Oxide–Containing Hydroxyapatite Coating in Antibacterial Cementless Total Hip Arthroplasty: Clinical Results of 50 Cases at 5-Year Follow-Up. Arthroplast. Today.

[184] Morimoto T., Hirata H., Eto S., Hashimoto A., Kii S., Kobayashi T., Tsukamoto M., Yoshihara T., Toda Y., Mawatari M. (2022). Development of Silver-Containing Hydroxyapatite-Coated Antimicrobial Implants for Orthopaedic and Spinal Surgery. Medicina.

